# Evaluating site selection for optimal photovoltaic installations and CO₂ emission reduction in selected districts of khyber pakhtunkhwa

**DOI:** 10.1038/s41598-025-86713-5

**Published:** 2025-02-24

**Authors:** Farnaz, Narissara Nuthammachot, Rabia Shabbir

**Affiliations:** 1 Faculty of Environmental Management, Prince of Songkla University, Hat Yai, 90110 Songkhla Thailand; 2 Department of Environmental Sciences, Fatima Jinnah Women University, Rawalpindi, Pakistan

**Keywords:** Photovoltaic (PV) solar installations, Multi-criteria decision-making (MCDM), ArcGIS Pro, Land use and land cover (LULC), Solar energy suitability, Geospatial analysis, Climate sciences, Environmental sciences, Energy science and technology

## Abstract

As the global market for renewable energy solutions expands, geospatial analysis is becoming crucial for optimizing solar potential. The current study assesses the suitability of installing PV solar system in the Mardan, Peshawar, and Nowshera districts in Pakistan using a multi-criteria decision-making (MCDM) approach. Analysis of different parameters, such as topography, land use and land cover (LULC), solar radiation and land surface temperature (LST), were performed to find the appropriate locations for solar in their respective regions. The study employed binary classification and weighted overlay methods to detect patterns of spatial suitability. Peshawar showed maximum ability with 859.8 km² categorized as favorable with a projected annual power output capacity of 67.77 trillion kWh and a decrease in CO₂ emission of 2.78 billion metric tons. Mardan closely followed the suitable area with 828.4 km² with energy generation of 39.74 trillion kWh/year and reduction of CO₂ emissions by 1.63 billion metric tons. Nowshera has an appropriate area of 503.0 km², for energy output of 670.06 billion kWh, and CO₂ reduction of 27.46 million metric tons. These results underline the importance of combining geospatial and meteorological data for accurate planning of solar energy systems. By highlighting location-specific features, including topography and solar irradiance illustrates the importance of tailoring energy outputs and environmental impacts to local contexts. These insights help guide policymakers in driving renewable energy projects, and support Pakistan’s sustainable development and climate targets.

## Introduction

The increasing population and urbanization in Pakistan have led to a significant rise in electricity demand, creating a growing gap between supply and demand^1,2^. In addition, the energy shortage is compounded by excessive inefficiency in energy generation, obsolescent infrastructures and an overdependence on fossil fuels^3^. This gap between supply and demand results in severe electricity shortages across Pakistan, leaving approximately 45 million individuals without access to electric lights, and many more facing frequent outages. The provision of reliable electricity is ranked at 110 out of 135 countries in terms of power sector performance and according to the World Bank, inadequacies in the system cause an economic loss equal to 6% of GDP^4^. The country’s installed capacity of approximately 23,600 MW consistently falls short of peak demand, creating a shortfall of 3,000 to 6,000 MW. As of 2023, Pakistan’s energy mix heavily relies on gas and coal, contributing 34.14% and 17.99%, respectively. In contrast, renewable sources such as hydropower and solar remain underutilized, constituting 23.53% and only 0.9%, respectively^5^. This dependence on carbon-based fuels not only exacerbates environmental challenges but also contributes significantly to CO₂ emissions, which account for nearly 60% of Pakistan’s greenhouse gas emissions^6,7^. Its environmental impact differs significantly as coal is responsible for around 1.4–3.6 pounds CO_2_e/kWh, whereas natural gas has from 0.6 to 2.0 pounds CO_2_e/kWh. In contrast, alternative renewable forms of energy, such as solar, have from 0.07 to 0.20 pounds CO_2_e/kWh^8^. The trend towards increasing dependence on non-renewable energy sources has worsened environmental issues and highlights the need for cleaner alternatives like solar energy to complement the growing electricity demand^9^. Given the stark environmental and supply challenges posed by current energy sources, transitioning to cleaner forms of energy is becoming an inevitable necessity.

In contemporary society, photovoltaic systems have become more and more popular in the generation of electricity. The general deriving perspective is favorable, as all coal-created electricity can be substituted with only 3% of the PV systems that would be equipped on all appropriate surfaces^10^. Being one of the most important sources of renewable energy, solar power has the potential to cover 40% of the electricity demand in the United States, as projected by the US Department of Energy’s Solar Energy Technologies Office and the National Renewable Energy Laboratory^11^. Power generation is provided by photovoltaic or PV systems that convert sunlight directly into electricity, and its advantage is the conversion of solar energy into eco-friendly, clean electricity^12^. The most effective ones include stand-alone, grid-connected, hybrid, and building-integrated panels^13^. The installation of PV systems should be cost-effective for both energy consumers and providers. While these trends in solar energy are rapidly unfolding globally, Pakistan’s reliance on non-renewable sources underscores the need for a shift towards solar power.

Despite possessing vast solar energy potential—estimated at over 100,000 MW—Pakistan has been slow in adopting solar technologies. The country’s untapped geographical advantages, particularly in regions like Mardan, Peshawar, and Nowshera, with high solar irradiance, offer an opportunity to bridge this gap. While Solar energy has experienced rapid growth worldwide (with leading contributions from China and the United States ), with a combined global installed capacity of 1,177 GW by the end of 2022^14,15^, Pakistan is not actively participating in this energy transition. Pakistan has significant solar energy resources, with potential estimated at 2,900,000 MW and over 300 days of sunshine per year generating 5.5–6 kWh/m²/day^16^. Nonetheless, despite these natural advantages, its share in electricity is still insignificant^17^.The projects like the Quaid-e-Azam Solar Power Park in Pakistan are a good starting point spearheaded by the government, yet a lot more is required to bridge this gap in energy demand and supply on the micro as well as macro level^18^. Enhanced research, development, and supportive policies are crucial for promoting solar energy adoption^19^.

Several studies have explored the potential of solar energy in Pakistan, but most are limited to solar irradiance or the economic aspects of solar projects. For instance, [6] highlight the significant carbon intensity in Pakistan’s power sector due to thermal energy reliance, emphasizing the need for cleaner technologies. However, their study does not incorporate geospatial analysis or decision-making frameworks critical for optimizing site-specific renewable energy deployment. Similarly, while the study on off-grid solar pv power generation in sindh^20^analyzes the techno-economic feasibility, it does not consider the trade-off associated with land use and site suitability from an environmental perspective. Likewise, the ahp-fuzzy vikor method in solar pv site selection in pakistan^21^despite presenting an innovative method, does not consider the integration with local climatic data like land surface temperature (LST) and land cover/land use changes. Similarly, the SWOT-AHP and fuzzy-TOPSIS approach assessed strategies for sustainable energy planning in pakistan^22^, but does not provide a comprehensive analysis of site-specific characteristics that are likely to affect solar energy deployment (such as solar irradiance, slope, aspect, lulc changes).

This research aims to cover the gap by assigning potential sites and development toward energy efficiency among photovoltaic (PV) deployment in Mardan, Peshawar, and Nowshera. With 4.3 kWh/m² of mean solar radiation throughout the year and large areas of sunshine, Pakistan holds great potential for solar energy, which could help meet its growing energy deficit^4^. This research combines geospatial tools and multi-criteria analysis to contribute towards a scientific platform for rationally extracting the best locations close to large-scale settlements based on CO₂ reduction with economic gain in deploying solar infrastructures.

Based on previous studies that focused on evaluating the effects of land use/land cover (LULC) changes and the surface temperature rises in Peshawar, Mardan, and Nowshera, the findings in this research will be utilized for identification of the best eligible site for photovoltaic (PV). We provide a holistic response to climate through inclusion of new LULC data along with results from renewable energy studies. This study emphasises the two-way role of renewable energy by combating LULC change environmental impacts while also facilitating sustainable energy solutions to the energy crisis challenge Pakistan is facing. It points to the future of PV installations to develop an environmental resilience as well as energy sustainability.

The primary objective of this research is to evaluate the optimal site selection for PV solar installations by analyzing parameters such as solar irradiance, slope, aspect, viewshed, geomorphic landforms, LULC, land surface temperature (LST), and hillshade. These parameters were synthesized using the weightsum tool in ArcGIS to identify the most suitable locations for PV deployment. Each of these factors plays a critical role in determining the potential for energy production and environmental suitability: for example, slope and aspect influence solar exposure, viewshed analysis prevents shading, and LULC and LST account for environmental conditions that affect performance. This multi-criteria analysis allows for a holistic assessment, offering a detailed understanding of both the environmental conditions and energy output potential for each site. The outcomes of this study support Pakistan’s broader energy transition goals, particularly its commitment to increasing renewable energy’s share to 30% by 2030 while simultaneously promoting environmental sustainability and economic growth.

Globally, the transition toward renewable energy, particularly solar power, reflects a growing recognition of the urgent need to reduce reliance on fossil fuels. Although solar photovoltaic (PV) systems have achieved a global installed capacity of 1,177 GW by 2022 has immediately influenced their accession into the power relay across significant countries, including China and The US, which have been among the state’s leading world economies^14,23^. Pakistan lags in the energy transition due to socio-economic conditions, regulatory impediments, and an obsolete electricity grid, further exacerbated by a high reliance on fossil fuels^19,24^. Known to hold only a mere 5.4% share of the energy production, predominantly from biomass, wind, and sun power in Pakistan; however, there seem to be countless promises that need to be made. Pakistan has declared to raise renewable energy use by another 30% till the upcoming Decade (2030)^17^and achieving these targets is obligatory only if there exists a mix of pragmatic policy change, technological breakthroughs with nice site selection for way broader deployment like PV installations^19,25,26^.

The barriers to solar energy adoption in Pakistan can be overcome through a multi-faceted approach involving policy overhaul, tech upgrades and community buy-in. Partnership and collaboration between the government, private sector, and local communities are essential in securing resources to finance renewable energy projects^24^. This work highlights the need for parameters set to maximize PV installation sites that can inform policy that decreases CO_2_ emissions and improves energy efficiency. In addition, it said encouraging the use of solar power in places such as agriculture could vastly increase farmers’ income, proving that a move to cleaner energy is also economically beneficial^27^. In sum, through the integration of local and broad policy considerations, this research is gaining insights that will help Pakistan realize national and global sustainability targets.

Past studies in the solar energy realm have shown that site selection is essential to maximize power production and minimize environmental impact. Systematic evaluations of different sites by incorporating environmental, economic and social aspects are essential to boost solar PV deployment using tools as Analytic Hierarchy Process (AHP) or Geographic Information Systems (GIS)^28^. These analyses should account for the importance of factors such as solar irradiance, topography, land cover and local climate conditions^29,30^. For example, slope and aspect are critical to solar panel placement^31^, whereas view shed analysis allows the planners to know her site is not shaded. The use of geomorphic landforms (LST) and hillshade analysis provides further granularity by incorporating both the physical properties as well as thermal qualities of the landscape into the caret selection process^32,33^.

In Pakistan, site suitability analyses with respect to PV installations are rarely conducted, and these mostly emphasize only solar irradiance. This research fills this gap by incorporating various geospatial parameters for the analysis of Mardan, Peshawar, and Nowshera. In this analysis, a weight sum tool (ArcGIS-based multi-criteria decision-making approach) amalgamates solar irradiance, slope, aspect, viewshed, LULC, etc. Parameters to identify the best suitable areas for installing a solar power plant. The validity of this approach has been tested in prior research^28,30,34^, that found improved performance for identifying sites suitable to host renewable energy projects^24,34,35^.

There is an increasing acknowledgement of the need for integrating LULC and climate, particularly when it comes to renewable energy planning, which often results in sustainable development. The previous studies conducted in Peshawar, Mardan, and Nowshera have indicated marked changes in LULC during the last few decades, especially due to urban encroachment coupled with deforestation, which in response has resulted in land surface temperature. Through leveraging the science of land change, which includes LULC data outside of just project sites for energy studies, this paper covers issues related to environmental consequences and remedies by focusing on renewable energy solutions within a larger context.

In sum, the results of this research are relevant to essential policy. Advances in solar technology have led to a wide range of indoor and outdoor applications, but the fundamental importance of where we put PV remains. This knowledge will contribute to lowering the CO₂ emissions as well as facing electricity issues, which is also aligned to sustainable development goals (SDGs) of Pakistan under UN SDG 7 (Affordable and Clean Energy) and UN SDG 13(Climate Action). The results provide an evidence-based framework for the realization of Pakistan’s renewable energy ambitions, which are able to deliver both economic and environmental sustainability.

The primary objectives of this research are:

1. Evaluate and identify the most suitable locations for photovoltaic (PV) solar installations in Mardan, Peshawar, and Nowshera using geospatial and multi-criteria analysis tools.
2. To assess the energy efficiency and potential CO₂ emissions reduction associated with different site selections for PV installations.
3. Offer practical recommendations for local governments and energy planners to enhance the adoption of renewable energy and support Pakistan’s renewable energy goals in alignment with the United Nations Sustainable Development Goals (SDGs).

## Study area

**Fig. 1:**
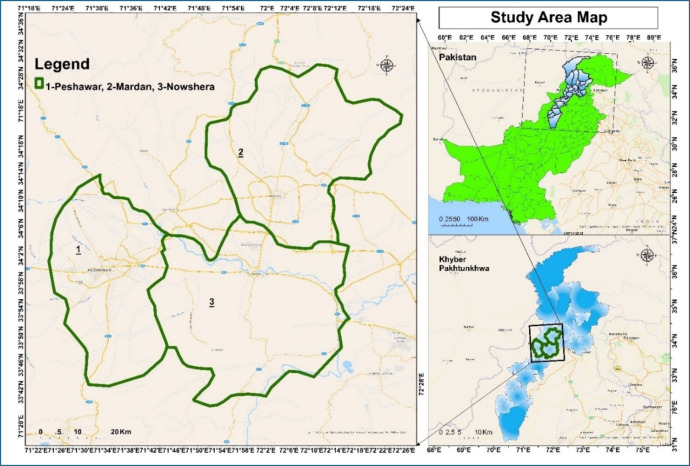
Geographic and Climatic Overview of Peshawar, Mardan, and Nowshera.

The study investigates Peshawar, Mardan and Nowshera (Fig. 1) districts situated in Khyber Pakhtunkhwa province of Pakistan supposed to be most potential for photovoltaic solar installations due to variation with respect to their geographical and climatic features.

The capital of Khyber Pakhtunkhwa is Peshawar (34.0151° N, 71.5249° E). The city is southwest of Islamabad-Pakistan and east-southwest of Kabul–Afghanistan, located in the Peshawar Valley. There is a mix of flat plains and gentle hills in this region, and land use includes urban residential, commercial retail areas, and industrial zones. Peshawar has a semi-arid climate characterized by high temperatures, with many hot summer days well above 40 °C (104 °F), and mild winters with moderate day time maximum averaging between 5 °C and 15 °C also in night low/frosts are common. (41 °F to 59 °F) Additionally, solar energy technologies are a good fit in the region due to its high level of solar irradiance.

Mardan altitude is about 34.1887° N latitude and 72.0517° E longitude. It is flat with gentle undulations in the middle of Khyber Pakhtunkhwa(topic). It is mostly occupied by thousands of farms, which rely heavily on the local geomorphology for agricultural land use. Mardan has a semi-arid climate with hot summers; temperatures in summer can be over 40 °C (104 °F) and winter normally also between 5 and 15 degrees Celsius (41–59 Fahrenheit). The plentiful farmland in Mardan accompanied with the high solar irradiance levels make up a huge opportunity for effective use of solar power.

Nowshera, situated at around 34.1161° N latitude and 73.2016° E longitude is located along the Kabul River of Khyber Pakhtunkhwa in eastern edit Pakistan Make Your Way Through The Popular Posts The district consists largely of flat river plains with a few low hills. It is characterized by a semi-arid climate: hot summers, with frequent temperatures of 40 ° C or more (104 ºF), and mild winters, generally in the range between 5 °C to15°C. It is situated in the urban, industrial, and agricultural zone of Nowshera. With its top ranking of solar irradiance, it can be a feasible site for future developments in the area and, therefore, support overall sustainability efforts due to renewable energy generation.

The same semi-arid climate and high solar irradiance in all three districts of Peshawar, Mardan, and Nowshera are two principal determinants for a successful photovoltaic installation^24,36,37^. The information collected as part of this strategic assessment will help us better plan for placement in each location, to increase energy reliability and reduce both CO₂ emissions across those regions.

### Methodology

#### Data collection

For this study, a comprehensive set of datasets was gathered to analyze the optimal locations for photovoltaic (PV) solar installations in Mardan, Peshawar, and Nowshera. The following datasets were used:

Digital Elevation Models (DEMs): DEMs were acquired from the USGS Earth Explorer portal (https://earthexplorer.usgs.gov). This information was required to perform the calculations for slope, aspect, hillshade, viewshed, and landforms. The DEM provided the topographic information necessary for the analysis to determine the suitability of the target site for the installation of a photovoltaic power plant.

Land Use/Land Cover (LULC) Data: The Sentinel-2 imagery was sourced from the Copernicus Open Access Hub. It was processed and classified using the RF algorithm to distinguish between different LULC classes. These data were useful in determining the suitability of land for PV installation.

Land Surface Temperature (LST): Landsat 9 images were retrieved from the USGS Earth Explorer portal for the proposer’s calculation purposes. There are several steps in this process. These include the calculation of the Top-of-Atmosphere reflectance for bands 4 and 5 – which are the red and near-infrared – followed by the establishment of the NDVI from these two bands. This activity was then performed to estimate the overall surface emission from the categories and sub-categories. The fourth part was to derive the brightness temperature from Band 10, which is the Thermal Infrared. Finally, these components of the estimation were used in the predetermined formula for the LST calculation.

## Data processing

This section describes the data processing methods which were used in this study to determine the PV suitability of different areas. DEMs, Sentinel 2 images, and Landsat 9 images, and other types of datasets were processed to derive critical parameters like terrain condition, land cover, and land surface temperature that are crucial for considering the solar power potential.

## DEM data processing

In this study, the Digital Elevation Models (DEMs) were meticulously processed within ArcGIS Pro to assess photovoltaic (PV) suitability. DEM rasters were clipped with respect to the study areas of Peshawar, Mardan, and Nowshera. Key terrain parameters, such as slope and aspect, were used to understand their effects on solar irradiance/ PV efficiency. These were combined, and areas exposed to the sun with no shadow-casting elements in viewshed analysis — or vice versa for heat island prioritization maps. In addition to this alluvial belt, geomorphic landforms were classified using this method based on their potential impact on PV installation suitability. This exhaustive pre-processing will give adequate insight into the potential of any area, which includes topographic as well as environmental influences over suitability mapping for PV.

## Sentinel-2 imagery processing

Land Cover classification of the processed Sentinel-2 imagery (2023 ) in 5 classes was done using the RFA algorithm and QGIS. The five different categories of land were Water, Builtup, Barren, Forest/Vegetation, and Farmland. This allowed us to create binary maps of the classifications and consequently divide PV compatibility into classes (i.e., specific regions either suitable or unsuitable for potential PV installations). This reclassification allowed a fairer comparison and aggregation of disparate measures.

### Random forest algorithm

Random Forest Algorithm (RFA) was used to classify the processed Sentinel-2 imagery into five landcover types (Table 1). Random Forest (RFA) is an ensemble learning method that builds many decision trees when training and outputs the class mode of the individual trees as the classification outcome^38^.By utilizing a subset of the training data to build each tree in the forest it reduces overfitting and helps improve the accuracy of the classification^39,40^. Using this strategy, RFA can efficiently process extensive and sophisticated datasets, identifying complex patterns and correlations in remote sensing images. This is an appropriate method for the study area because it gives a precise and high confidence classification of land cover classes to be used^41^ for assessing areas of suitability for photovoltaic (PV) deployment.

**Table 1:** Classes delineated based on supervised classification.

Sr. No.	Class Name	Description
1	Farmland	Land used for growing crops or kept fallow for agricultural purposes.
2	Built-up Area	Areas dominated by human-made structures, including residential and commercial buildings, roads, and infrastructure.
3	Barren Area	Land with little or no vegetation, often due to human activity.
4	Vegetation/Forest Area	Areas covered by trees and other vegetation, including forests and grasslands.
5	Water	Surface water bodies such as rivers, lakes, ponds, and reservoirs.

## Landsat 9 imagery processing for LST calculation

Land surface temperature (LST) was modeled from Landsat 9 using a series of steps. Top-of-Atmosphere (TOA) reflectance was initially calculated using Band 4 (Red) and 5 Near-Infrared to compensate for other atmospheric influences. With these bands, the Normalized Difference Vegetation Index (NDVI) was obtained, from which surface emissivity can be estimated, and it is crucial to grasp land surface heat emission better. The actual brightness temperature was then attained using Band 10 (Thermal Infrared), allowing the thermal data needed to calculate LST. These following steps were necessary for the reliable LST estimation that is of critical importance to quantify the thermal environment at possible photovoltaic (PV) installation sites. The tutorial (step 1 to 6) provides the equations and steps to calculate the Top of the Atmosphere, which can be found in its Radiance, Emisivity, Brightness temperature, and NDVI Normalized Difference Vegetation Index.

Step 1: Calculate the top of the atmosphere spectral radiance. Equation 1

1 $$\:\begin{array}{c}L={M}_{L}\times\:{Q}_{cal}+{A}_{L}\end{array}$$

The formula converts the raw pixel values ($\:{\varvec{Q}}_{\varvec{c}\varvec{a}\varvec{l}}$) into radiance values by applying a linear transformation. The radiance values are essential for further processing and analysis, such as calculating surface reflectance or brightness temperature. The process involves multiplying the raw values by a gain factor ($\:{\varvec{M}}_{\varvec{L}}$ ) and then adding an offset (AL) to obtain the TOA radiance.

Step 2: Convert TOA to brightness temperature: Eq. 2

2 $$\:\begin{array}{c}BT=\left(\frac{K2}{Ln\left(\frac{K1}{L}\right)+1}\right)-273.15\end{array}$$

Step 3: calculate NDVI. Equation 3

3 $$\:\begin{array}{c}NDVI=\frac{NIR+RED}{NIR-RED}\end{array}$$

Step 4: Calculate the proportion of Vegetation Pv: Eq. 4

4 $$\:\begin{array}{c}Pv=\frac{NDVI-{NDVI}_{min}}{{NDVI}_{max}-{NDVI}_{min}}\end{array}$$

The $\:{NDVI}_{\varvec{m}\varvec{i}\varvec{n}}$ value, representing non-vegetated surfaces and $\:{NDVI}_{\varvec{m}\varvec{a}\varvec{x}}$ value, representing fully vegetated surfaces.

Step 5: Calculate emissivity. Equation 5

5 $$\:\begin{array}{c}\in\:=0.004\:\times\:\:Pv+0.986\end{array}$$

Step 6: Calculate LST: Eq. 6.

To calculate Land Surface Temperature (LST) from Landsat thermal infrared data is:

6 $$\:\begin{array}{c}LST=\frac{{T}_{B}}{1+\left(\frac{\lambda\:{T}_{B}}{\rho\:}\right)Ln\left(\epsilon\right)}\end{array}$$

Where $\:{T}_{B\:}$represents the brightness temperature in Kelvin, which is derived from the thermal band radiance measurements. The wavelength of emitted radiance, denoted by λ, is specific to the thermal band and is measured in meters. The parameter ρ is a constant value derived from Planck’s constant times the speed of light divided by Boltzmann’s constant, typically approximately 1.4388 × 10^4 m·K. Finally, signifies the emissivity of the surface, which accounts for the surface’s ability to emit thermal radiation. The derived Land Surface Temperature (LST) maps for the three districts of Peshawar, Mardan, and Nowshera are shown in Fig. 3 (a, b,c).

**Fig. 2:**
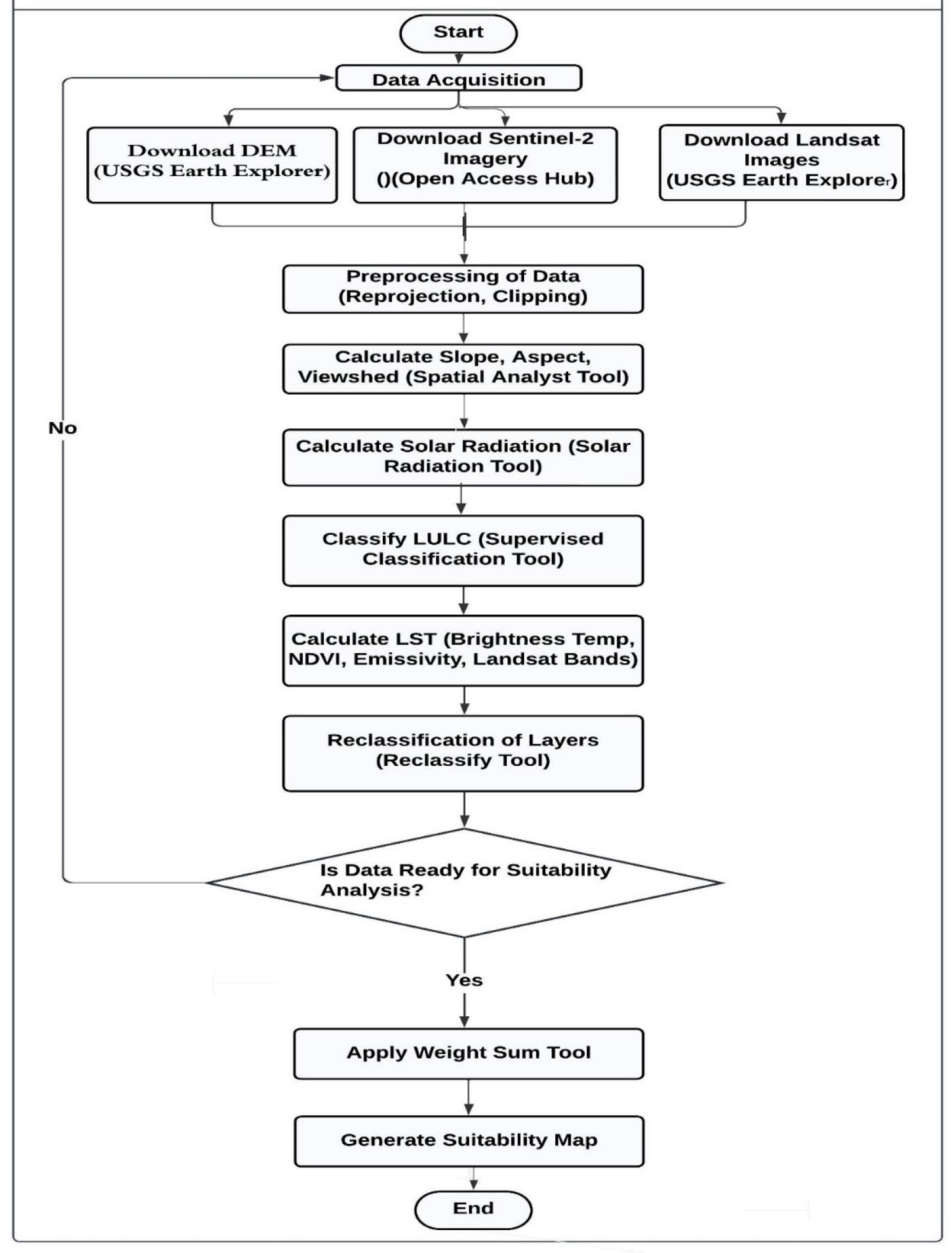
Workflow for Generating a Suitability Map for Photovoltaic Installations Using ArcGIS Tools.

## Reclassification and suitability analysis

Once all parameters (slope, aspect, viewshed, solar radiation, geomorphic landforms, land use/land cover (LULC), and land surface temperature (LST)) were derived, they were reclassified into binary values (0 for unsuitable and 1 for suitable) using the Reclassify tool in ArcGIS Pro (Tables 2, 3, 4, 5, 6, 7, 8 and 9). This reclassification transformed continuous data into categorical classes based on predefined thresholds, making it easier to analyze and interpret the suitability of different areas for photovoltaic (PV) installations.

The multi-criteria decision-making(MCDM) method was applied to assess and ranking these parameters for their relevance to the pv installation suitability. The specification used is elaborated in heading “weight sum analysis for pv site suitability”, where a weighted sum model (wsm) was implemented to aggregate the normalized parameters into a single suitability score.

**Table 2:** Reclassification of Solar Radiation (a-Mardan, b Nowshera, c Peshawar).

Start Value	End Value	Binary Value
(a)		
286.31	1050.61	0
1050.61	1233.13	0
1233.13	1352.91	1
1352.91	1444.16	1
1444.16	1740.76	1
(b)		
206.52	954.77	0 (not suitable)
954.77	1197.95	0 (notSuitable)
1197.95	1347.60	1 (Suitable)
1347.60	1453.60	1 (Suitable)
1453.60	1796.54	1 (Suitable)
(c)		
647.999023	1185.912454	0
1185.912454	1338.563563	0
1338.563563	1385.812715	1
1385.812715	1411.254567	1
1411.254567	1574.809326	1

**Table 3:** Reclassification of Hillshade (a - Mardan, b- Nowshera, c- Peshawar).

Start Value	End Value	Binary Value
(a)		
0	80	0 (Not Suitable)
80	142	0 (Not Suitable)
142	178	0 (Not Suitable)
178	207	1 (Suitable)
207	254	1 (Suitable)
(b)		
0	98	1
98	147	1
147	176	1
176	202	0
202	254	0
(c)		
0	141	1
141	169	1
169	178	1
178	185	0
185	251	0

**Table 4:** Reclassification of LST (a-Mardan, b- Nowshera, and Peshawar).

Start	End	Binary
(a)		
−1.52409	2.211582	0
2.211582	4.318885	0
4.318885	5.276749	0
5.276749	6.3304	1
6.3304	10.68868	1
(b)		
−0.408567	2.949274	0 (Not Suitable)
2.949274	3.85192	0 (Not Suitable)
3.85192	4.537931	0 (Not Suitable)
4.537931	5.296153	1 (Suitable)
5.296153	8.798418	1 (Suitable)

**Table 5:** Reclassification of slope (Mardan, Nowshera, Peshawar).

Start Value	End Value	Binary Value
0.00	1.72	1 (Suitable)
1.72	3.43	1 (Suitable)
3.43	5.71	1 (Suitable)
5.71	8.53	1 (Suitable)
8.53	11.3	1 (Suitable)
11.3	14.04	0 (Not Suitable)
14.04	16.7	0 (Not Suitable)
16.7	21.8	0 (Not Suitable)
21.8	30.96	0 (Not Suitable)
30.96	45.00	0 (Not Suitable)
45.00	90.00	0 (Not Suitable)

**Table 6:** Reclassification of Aspect (Mardan, Nowshera, Peshawar).

Start Value	End Value	Direction Description	Binary Value
−1	−1	Flat	0
−1	22.5	North	0
22.5	67.5	Northeast	0
67.5	112.5	East	0
112.5	157.5	Southeast	1 (Suitable)
157.5	202.5	South	1 (Suitable)
202.5	247.5	Southwest	0
247.5	292.5	West	0
292.5	337.5	Northwest	0
337.5	360	North	0

**Table 7:** Reclassification of Viewshed ( Mardan, Nowshera, Peshawar).

Start Value	End Value	Binary Value
0	0.9	1 (Suitable)
0.9	1.8	1 (Suitable)
1.8	2.7	1 (Suitable)
2.7	3.6	1 (Suitable)
3.6	4.5	0 (Not Suitable)
4.5	5.4	0 (Not Suitable)
5.4	6.3	0 (Not Suitable)
6.3	7.2	0 (Not Suitable)
7.2	8.1	0 (Not Suitable)
8.1	9	0 (Not Suitable)

**Table 8:** Reclassification Table for Geomorphic Landforms: (Mardan, Nowshera, Peshawar).

Start Value	End Value	Landform	Binary Value
1	1	Flat	1
2	2	Peak	0
3	3	Ridge	0
4	4	Shoulder	0
5	5	Spur	0
6	6	Slope	0
7	7	Hollow	0
8	8	Footslope	0
9	9	Valley	0
10	10	Pit	0

**Table 9:** Reclassification of LULC (Mardan, Nowshera, Peshawar).

LULC Classification	Binary Value
Water	0 (Not Suitable)
Development	0 (Not Suitable)
Barren	0 (Not Suitable)
Forest/Vegetation	0 (Not Suitable)
Farmland	1 (Suitable)

The reclassified parameters were integrated into a suitability analysis, allowing for the identification of locations most conducive to PV installations. The following sections detail the reclassification and thresholds used for each parameter in the Mardan, Nowshera, and Peshawar districts.

### Selection of thresholds for site suitability in photovoltaic installations: justification and criteria

In this study, several factors were considered to determine the optimal sites for photovoltaic (PV) installations in Mardan, Peshawar, and Nowshera. Each factor was carefully selected based on its relevance to solar energy generation and its environmental, geographical, and economic implications. The factors include solar radiation, hillshade, land surface temperature (LST), slope, aspect, viewshed, geomorphic landforms, and land use/land cover (LULC), each of which was reclassified into suitable and unsuitable categories based on established thresholds.

### Solar radiation

The threshold for solar radiation was selected based on the solar energy potential of the region, as outlined by^42^ who assessed the spatial and temporal distribution of solar energy and its suitability for photovoltaic development.Solar radiation is the most essential parameter for energy production from PV systems, and regions with abundant solar radiation are more favorable for solar energy generation. From Solar radiation values for Mardan, Nowshera and Peshawar were categorized into two processes. Radiation values lower than 1197 W/m² were identified as not suitable areas as they provide insufficient energy for efficient PV operation, while radiation values between 1197 W/m² and 1796 W/m² were ranked as suitable areas due to their high solar energy potential Table 2 (a, b, c).

### Hillshade

Hillshade was used to represent the amount of sunlight a surface receives based on its topography. Higher hillshade values indicate that the area is exposed to more direct sunlight, making it more suitable for solar installations^43^. For all three districts, areas with hillshade values above a certain threshold were deemed suitable, as they receive more sunlight, ensuring efficient energy capture for PV panels. Locations with low hillshade values, indicating areas that are shaded for most of the day, were considered unsuitable due to their reduced exposure to sunlight. The Hillshade tool in ArcGIS Pro was used to generate the hillshade map, which was then reclassified based on the sunlight exposure values^44^, with areas receiving values from 178 to 254 classified as suitable and areas below this range classified as unsuitable (Table 3).

### Land surface temperature (LST)

The land surface temperature (LST) was another key factor influencing site suitability. PV panels are more efficient in areas with moderate temperatures. High temperatures can reduce the efficiency of solar panels, while very low temperatures may lead to suboptimal energy production. Based on this understanding, areas with LST values between 4.5 °C and 5.3 °C were classified as suitable, as these temperatures are optimal for energy production. Values outside this range were deemed unsuitable because they either exceed the ideal temperature for PV efficiency or fall below the threshold for effective energy generation (Table 4).

### Slope

The slope of the terrain was an important factor in evaluating the suitability for photovoltaic (PV) installations^45^. Steep slopes present challenges for solar panel installation, primarily due to the high costs of construction, difficulties in stabilizing the panels, and the reduced stability of the terrain^46,47^. In this study, the threshold for slope was determined based on the accessibility and ease of installation of solar panels. Areas with slopes of up to 14.04° were classified as suitable for PV installation, as they provide an optimal balance of accessibility and stability, ensuring cost-effective construction and better solar exposure. Slopes exceeding 14.04° were classified as unsuitable due to the increased difficulty and cost associated with installation, along with potential concerns regarding panel stability. Additionally, slopes above 30° were not considered suitable for solar energy generation, as the construction costs and logistical challenges would outweigh the benefits of solar exposure in these areas. Thus, areas with slopes in the range of 0° to 14.04° were regarded as suitable, while steeper slopes, particularly those above 14.04°, were considered not suitable for PV installation (Table 5). This threshold ensures that the selected areas are both economically viable and provide an optimal environment for solar energy production .

### Aspect

The aspect of the land was considered because it determines how much sunlight a location receives throughout the day. South-facing slopes, or in some cases east-facing, are the most exposed to sunlight and therefore the most suitable for solar panels^31^. In this study, areas with aspects in the range of 112.5° to 202.5° were classified as suitable, as they offer the most sunlight exposure. Locations facing north or with steep angles were considered unsuitable, as they receive less direct sunlight, reducing their potential for energy generation Table 6.

### Viewshed

In this study, the viewshed analysis was conducted to evaluate the suitability of locations for solar energy production by assessing the amount of sunlight exposure an area receives throughout the day^48^. The Digital Elevation Model (DEM) of the study area was used as the primary data source for the analysis, providing elevation information to identify areas visible from specific observer locations. These observer locations, chosen for their high elevation, represent areas with good solar exposure. Using ArcGIS Pro’s Viewshed tool, the visibility from these points was analyzed by considering the surrounding terrain. The resulting viewshed map was then reclassified into two categories: suitable and unsuitable, based on threshold values that reflect the level of sunlight exposure. Areas with viewshed values between 0 and 3.6 were classified as suitable for photovoltaic (PV) installation, while areas with higher values, indicating reduced exposure to sunlight, were deemed unsuitable. This reclassified viewshed map was integrated with other factors such as solar radiation, slope, and land surface temperature (LST) to identify optimal sites for PV installations in Mardan, Nowshera, and Peshawar. By using the viewshed analysis, the study ensured that only locations with maximum sunlight exposure, free from shading by surrounding terrain, were selected for solar energy generation (Table 7).

### Geomorphic landforms

The geomorphic landforms of the region also played a significant role in determining site suitability. Flat landforms, such as plains or gently sloping terrain, are ideal for solar installations because they are easier and more cost-effective to develop. Areas with ridges, peaks, or other rugged landforms were classified as unsuitable because these areas are not only difficult to access but also may not receive sufficient sunlight due to their elevation or topographic features(Table 8).

### Land use/land cover (LULC)

Finally, land use/land cover (LULC) was considered to identify areas that are suitable for solar panel installations based on their current use^49^. Farmlands were considered the most suitable land use type, as they are typically open, flat, and accessible. On the other hand, areas covered by water bodies, urban developments, forests, or barren land were deemed unsuitable due to their inability to support solar panel installations or their poor exposure to sunlight (Table 9).

These tables provide a standardized approach to evaluating the suitability of land for photovoltaic installations across the three regions.

In conclusion, the thresholds for each factor were selected based on a combination of geographic, environmental, and economic considerations to ensure that the most suitable areas for PV installation were identified. These thresholds are grounded in the understanding of how each factor influences solar energy generation and reflects the characteristics of the region. By classifying each factor into suitable and unsuitable categories, this study provides a clear and systematic approach to identifying optimal locations for photovoltaic solar installations in Mardan, Peshawar, and Nowshera. The thresholds for each factor were further improved based on the Multi-Criteria Decision Making (MCDM) technique^1^. MCDM used knowledge from experts and local conditions to provide suitable weights for each factor, so that finally the thresholds for suitability classification were defined^50,51^. By integrating them into the MCDM process, it is assured that the thresholds are not only scientifically justified but also context-specific to local conditions of Mardan, Peshawar and Nowshera to guide the selection of suitable sites for photovoltaic^30,51^.

### Weight sum analysis for PV site suitability

After the binary classification, the Weighted Sum tool in ArcGIS Pro was employed to combine these parameters into a composite index of suitability^52^. The Weight Sum method is a multi-criteria decision-making (MCDM) technique that assigns weights to each parameter based on its relative importance in the site selection process^53^.Weights were then assigned to individual parameters based on their relative influence on photovoltaic (PV) suitability as arrive at by merging literature review with expert judgement^49^^53–55^.

Using the Weight Sum tool, a composite PV installation suitability map was generated by combining reclassified parameters and assigning corresponding weights based on their relevance to PV siting as depicted in Figs. 4, 5 and 6. The created maps can be used to identify the best places for PV solar installs. This approach helped identify optimal sites that maximize energy efficiency and minimize environmental impact, categorizing areas as suitable or unsuitable based on their weighted scores^56^.

Various studies^28,30,35,53,57^, suggest that the optimal weighting of parameters describes the best site selection for PV solar installations. For instance, solar radiation should receive a maximum weight of 25% as it is directly linked to the production output and efficiency of PV panels. Another significant parameter, land surface temperature, was weighted at 20% based on its impact on minimizing heat losses. Similarly, both slope and aspect should be given considerable weight, each of 15%, in an optimal site selection. The minimizing slope is critical for maximizing sunlight incidence, and the optimal aspect is essential in the northern hemisphere. Other parameters, viewshed, and land use land cover, should be included in the model to reflect the optimal weighting of 10% each based on considerations of maintaining areas with unobstructed solar access and adequate land use which previous studies found to be important. Finally, geomorphic landforms should receive the smallest weight, 3%, and hillsides should receive 2% as they are not critical in determining the suitability of a PV site. Table 10 below shows the weight scheme used for best combination.

**Table 10:** Weighting of parameters for optimal site selection of Photovoltaic (PV) solar installations.

Parameter	Weight (%)	Reason
Solar Radiation	25%	The most critical factor, as areas with higher solar radiation are ideal for PV efficiency.
Land Surface Temperature (LST)	20%	Significant because lower temperatures reduce heat losses, improving PV efficiency.
Slope	15%	Important for ensuring optimal sunlight incidence on panels; flatter areas are preferable.
Aspect	15%	Affects sun exposure; south-facing slopes are ideal in the northern hemisphere.
Viewshed	10%	Ensures the site has no obstructions, maximizing solar access.
Land Use Land Cover (LULC)	10%	Indicates land suitability; avoids urban or dense vegetation areas.
Geomorphic Landforms	3%	Minor factor affecting site stability and long-term viability.
Hillshade	2%	Minimal impact; identifies areas with terrain shadows affecting sunlight exposure.

In this study, we used a weighted sum model to combine multiple factors in determining the suitability of locations for photovoltaic (PV) installations. Each factor (such as solar radiation, slope, and aspect) is assigned a weight to reflect its relative importance in the analysis. The weighted sum formula is represented as: Eq. 7.

7 $$\:\begin{array}{c}W={\sum\:}_{i=1}^{n}Fi\times\:Wi\end{array}$$

Where F1, F2, and F represent different factors such as solar radiation, Slope, and aspect. W1, W2, and W ​ are the weights assigned to each factor. is the total number of factors considered in the analysis. This approach allows for the aggregation of spatial data based on their respective significance, helping to identify the most suitable areas for PV installation based on multiple environmental and topographical factors.

The weighted parameters are added together to form a single suitability score for that location. The output suitability map identifies the highest scoring areas as the most suitable sites for deploying PV. Areas that envisaged high scores for PV deployment are deemed attractive ones and low-scoring areas are established as less or even inappropriate for such infrastructures. Figure 2 shows the systematic workflow followed to create a suitability map for PV installations depicted in the diagram above, from data importingto analysis processes of ArcGIS analysis and overlay tools.

### General formula for area calculation

The total area is calculated based on the formula (Eq. 8) and given cell size for each region.

8 $$\:\begin{array}{c}\:\:\:\:\:\:\:\:\:\:\:\:\:\:\:Total\:Area\:\left(km^{2}\right)=\frac{Count\:\times\:(Cell\:size\:X\:\times\:Cell\:sixe\:Y)}{1000000}\end{array}$$

For area calculations, the cell sizes are as follows: Nowshera has a cell size of 29.99 m by 29.99 m, Mardan has a cell size of 63.51 m by 63.51 m, and Peshawar has a cell size of 29.98 m by 29.98 m.

### Combined suitability analysis using binary maps

We performed a combined suitability analysis by integrating binary maps to identify areas classified as suitable (1) or not suitable (0) based on the intersection of datasets. This was achieved using the Raster Calculator tool in ArcGIS with the formula: Suitability = Combined Binary Map = Con([Map1 = = 1] & [Map2 = = 1] & [Map3 = = 1], 1, 0). Figures 4, 5 and 6 illustrates the resulting map of combined suitability. Equation 9

$$\:\:\:\:\:\:\:\:\:\:\:\:\:\:\:\:\:\:\:\:\:\:\:\:\:\:\:\:\:\:\:\:\:\:\:\:\:\:\:\:\:\:\:\:\:\:\:\:\:\:\:\:\:Suitability={\sum\:}_{i=1}^{3}\left({Map}_{i}\times\:{W}_{i}\right)\:\:\:\:\:\:\:\:\:\:\:\:\:\:\:\:\:\:\:\:\:\:\:\:\:\:\:\:\:\:\:\:\:\:\:\:\:\:\:\:\:\:\:\:\:\:\:\:\:\:\:\:\:\:\:\:\:\:\:\:\:\:\:\:\:\:\:\:\:\:\:\:\:\:\:\:\:\:\:\:\:\:\:\left(9\right)\:$$

In this formula, $\:{Map}_{i}$​ represents the binary maps where areas suitable for photovoltaic installation are assigned a value of 1, and unsuitable areas are assigned a value of 0. The term $\:{W}_{i}$​ refers to the weight assigned to each map, reflecting the importance or influence of each factor in the suitability analysis. The summation ∑ aggregates the weighted contributions from each map, thereby calculating the overall suitability for photovoltaic installation in the study area. This method allows for the integration of multiple factors, where each factor is weighted according to its relevance. Figures 5, 6 and 7 illustrates the resulting map of combined suitability.

#### Energy output calculation

The energy output from the optimal PV installation sites was determined using Eq. 10, which factors in solar radiation and panel efficiency to calculate the total energy production.

$$\:{\:\:\:\:\:\:\:\:\:\:\:\:\:\:\:\:\:\:\:\:\:\:\:\:\:\:\:\:\:\:\:\:\:\:\:\:\:\:\:\:\:\:\:\:\:\:\:\:\:\:\:\:\:\:\:\:\:\:\:\:\:\:\:\:\:\:\:\:\:\:\:\:\:\:\:\:\:\:\:\:\:E}_{output\:\left(avg\right)}\text{}=A\left({\text{m}}^{2}\right)\times\:{I}_{avg}\:\times\:\eta\:\:\:\:\:\:\:\:\:\:\:\:\:\:\:\:\:\:\:\:\:\:\:\:\:\:\:\:\:\:\:\:\:\:\:\:\:\:\:\:\:\:\:\:\:\:\:\:\:\:\:\:\:\:\:\:\:\:\left(10\right)\:$$

Where $\:A$ represents the area in square meters, $\:{I}_{avg}$ is the average solar irradiance, and $\:\eta\:$ is the efficiency of the photovoltaic system. This formula provides a straightforward approach to estimate the energy produced by the system based on the available area, local solar conditions, and the efficiency of the PV panels. The values of suitable areas of all three districts, Peshawar, Mardan, and Nowshera are given in Table 7 (a, b, c).

### CO₂ emissions calculation

To assess the environmental impact, CO₂ emissions were estimated using Eq. 8. This involved applying the carbon footprint of solar panels and comparing it to the CO₂ emissions from traditional energy sources, providing a comprehensive evaluation of the environmental benefits of solar energy compared to conventional methods. The formula used for calculating CO2 emissions is Eq. 11 :

11 $$\:\begin{array}{c}CO_{2}=\frac{{E}_{used}}{{E}_{lifetime}\:\:\times\:{EF}_{grid}}\end{array}$$

where $\:{E}_{used}$​ refers to the total energy consumed (kWh), $\:{E}_{lifetime}$​ represents the total energy generated by the system over its lifetime (kWh), and $\:{EF}_{grid}$ is the grid emission factor, which quantifies the amount of CO₂ emitted per kWh of electricity generated from the grid (g CO₂/kWh).

## Results

This section involved geospatial analysis maps of the study region to analyze the suitability of installing solar photovoltaic (PV) in the Mardan, Nowshera, and Peshawar districts. It presents a series of maps illustrating the various determinants used for optimal solar site selection. All maps displayed significant information such as aspects, geomorphic landforms, hillshade, solar radiation surface/ terrain express LULC map layer, slope angle, and viewshed, and lastly, the outdoor temperature of the affected area. Therefore, this review provides a comprehensive analysis to identify suitable zones for harnessing sunlight energy through GIS base reconnaissance in these belts.

**Fig. 3:**
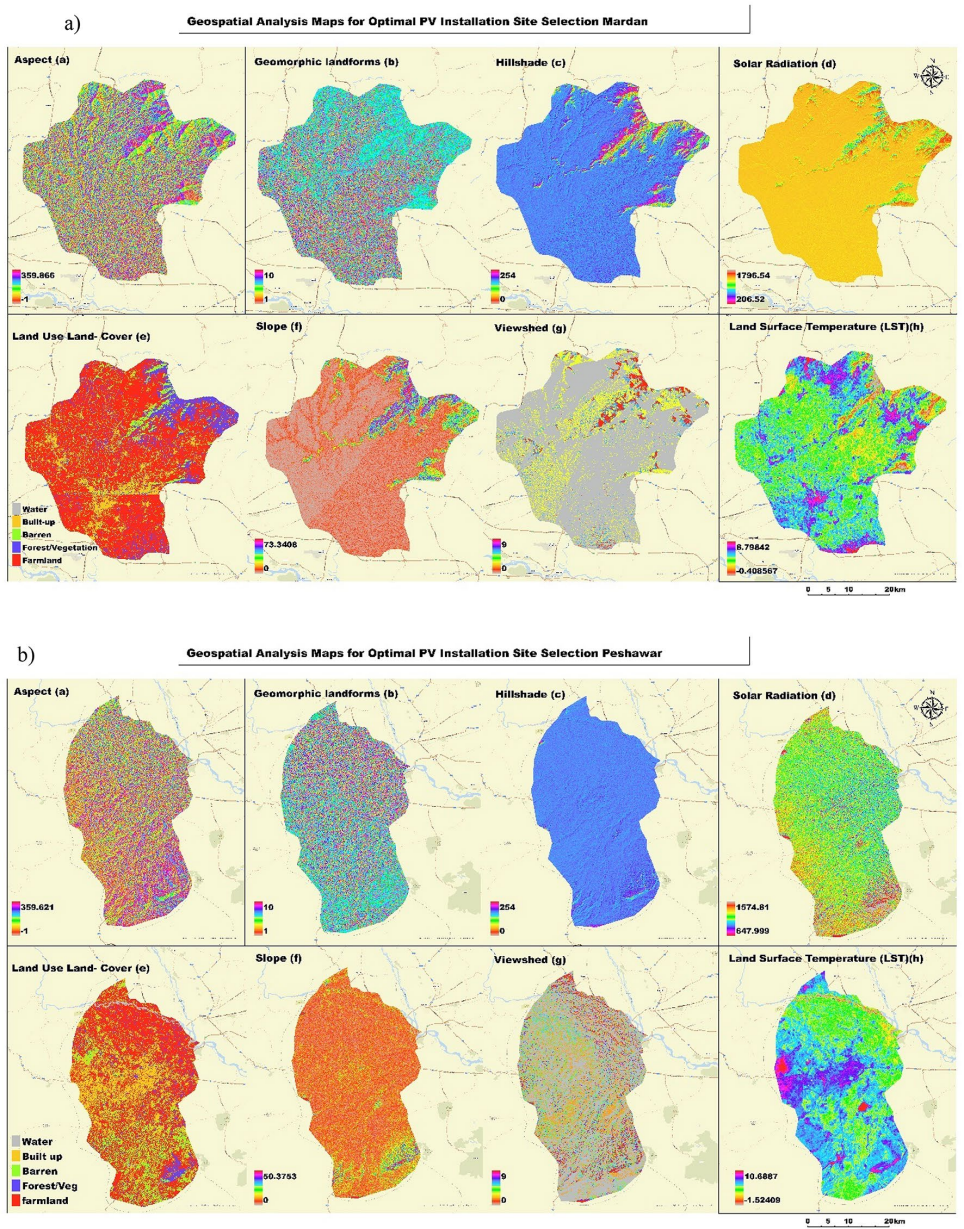

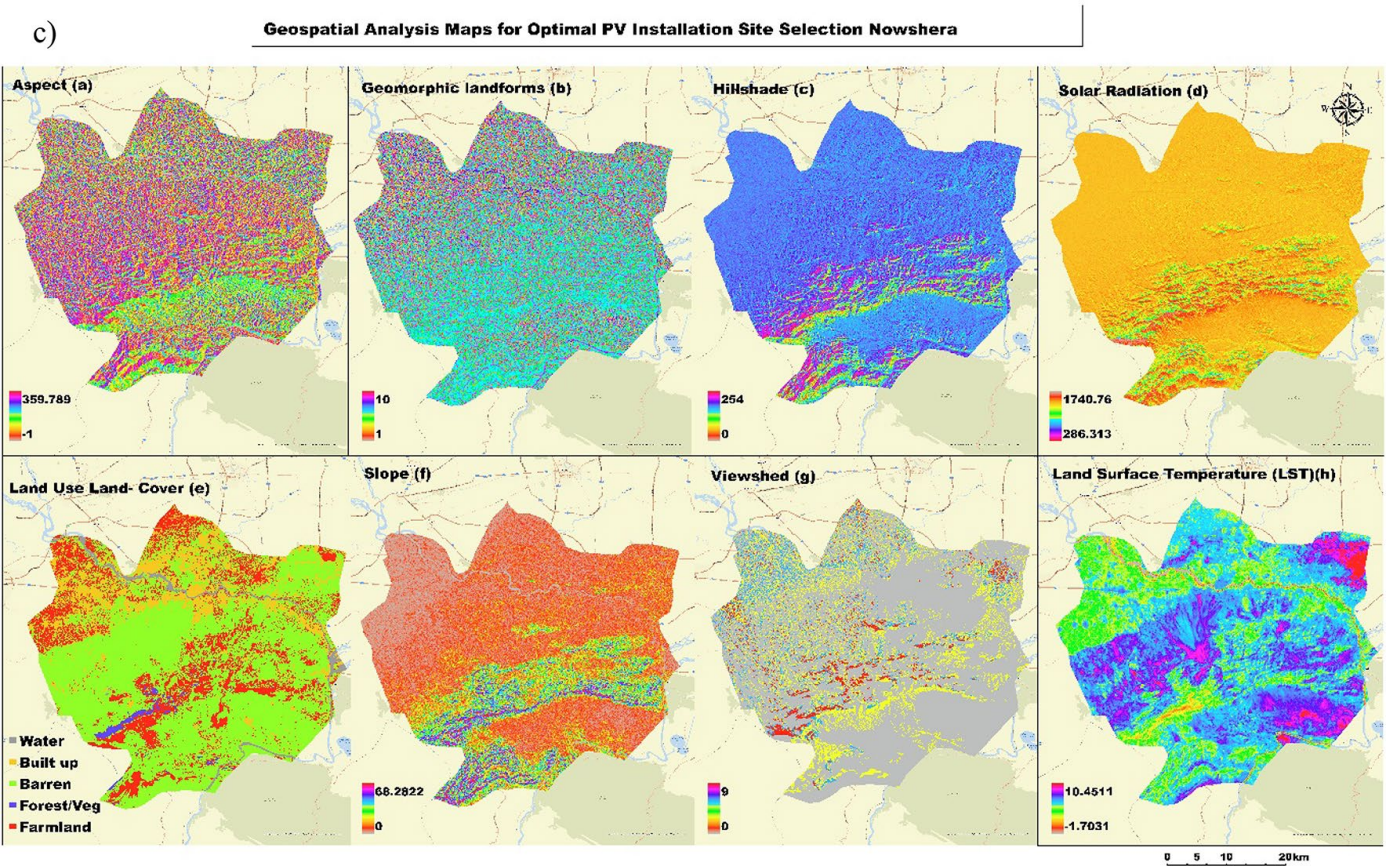
(**a**, **b**, **c**) Geospatial Analysis Maps for Photovoltaic Installation Suitability in Mardan (**a**), Peshawar (**b**), and Nowshera (**c**) Districts, Pakistan.

Figure 3a, b, and c is a full set of geospatial analysis maps to determine the best photovoltaic sites for study areas. It contains 8 maps, each depicting an important input to the suitability analysis. The first rows of maps highlight several important aspects related to the environment and topography: Map (a, b) depicts the slope aspect, which is necessary for determining solar exposition; The maps show geomorphic landforms, detailing the terrain’s physical features; Map (c) provides a hillshade visualization to highlight topographic relief; and Map (d) displays solar radiation distribution, with yellow areas indicating higher solar energy potential. The second row focuses on land characteristics and environmental factors: Map (e) classifies land use and cover into categories such as farmland, barren land, and forest, providing insight into current land utilization; Map (f) represents slope gradients, from low to high; Map (g) identifies visible areas from specific observation points, aiding in practical and aesthetic considerations for PV installation; and Map (h) shows land surface temperature variations, which can impact PV efficiency. In this comprehensive analysis, we provide a multi-scalar study that combines different datasets to give an in-depth evaluation of PV suitability considering natural and anthropogenic aspects for policymakers when making informed decisions on the development of solar energy in these regions.

**Fig. 4:**
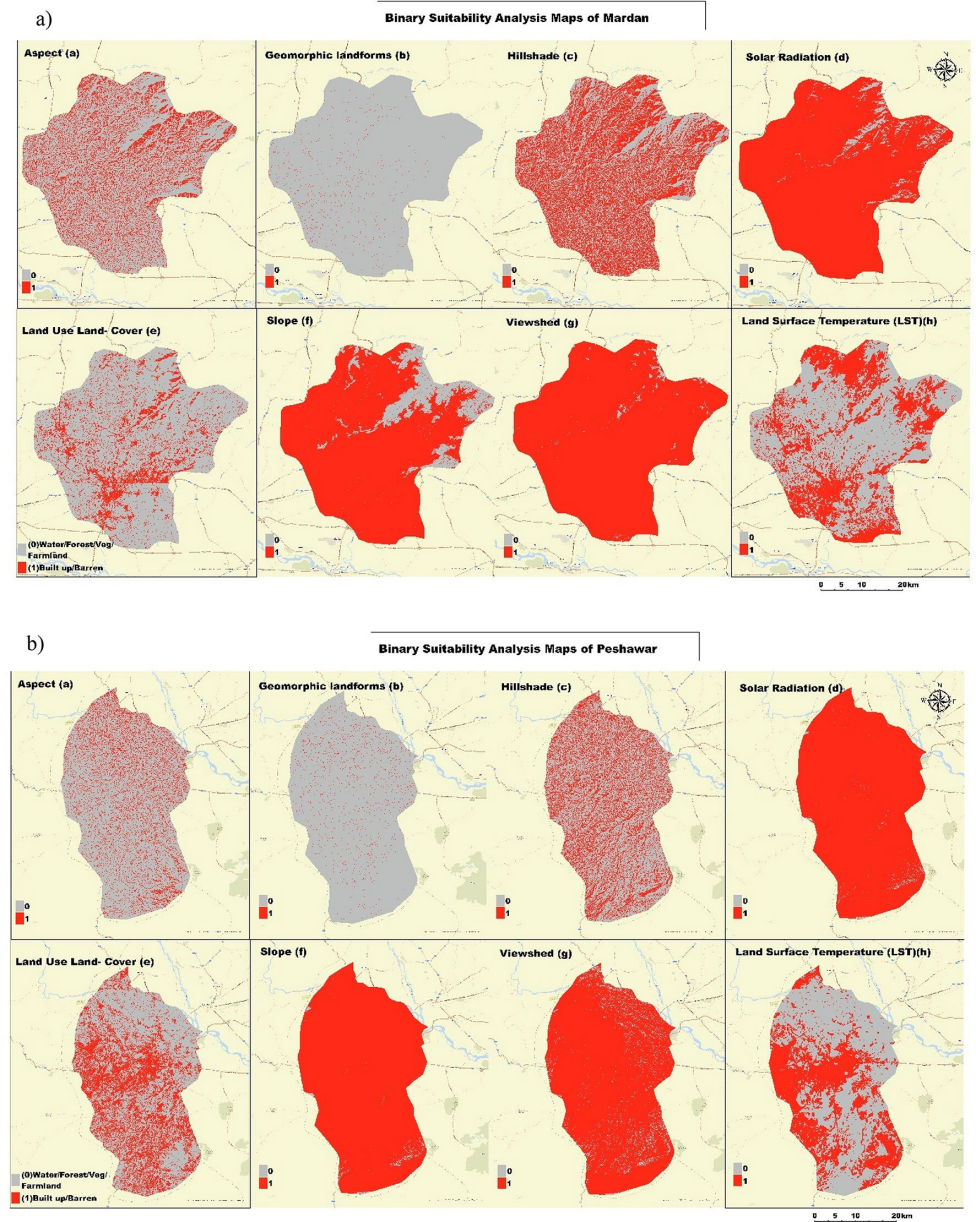

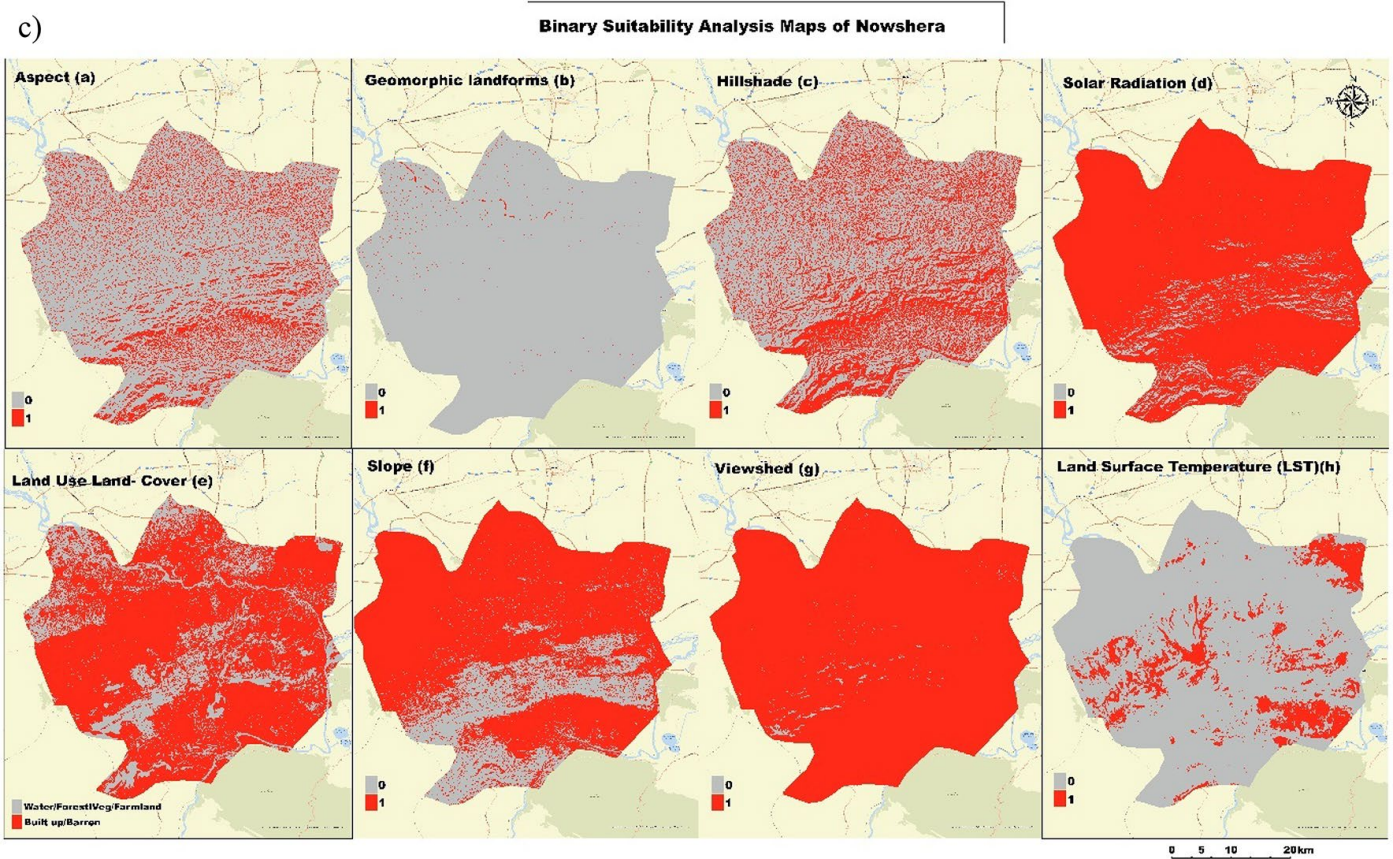
(**a**, **b**, **c**) Binary Suitability Analysis Maps for Photovoltaic (PV) Installation. Mardan (**a**), Peshawar (**b**), and Nowshera (**c**).

Figure 4a, b, and c present the binary suitability analysis maps for the Mardan district. These maps reclassify eight critical factors into binary categories: suitable (red) and not suitable (gray) for photovoltaic (PV) installation. These maps present the Mardan district, showing which areas meet the criteria for being suitable. In Mardan, Peshawar, and Nowshera, the same binary form of the Map shows which areas meet the criteria of photovoltaic installer across different factors. This consistent format across all three figures allows for a straightforward comparison of suitable areas for PV installation in the regions.

The presentation made it possible to navigate through all the factors influencing the suitability of installing PVs in Mardan, Peshawar, and Nowshera and to analyze them in relation to each other. Making the demand for the conditions of a particular situation known makes it a more straightforward concept to understand. The binary suitability maps allow you to form an immediate impression of where it is preferable to install PV and highlight areas where multiple positive conditions of overlap. That is one of the ways to identify sites with optimal conditions for the development of solar energy sources.

### Suitability analysis for photovoltaic installations in Mardan, Nowshera, and Peshawar district

This section presents a detailed suitability analysis for photovoltaic (PV) installations in the districts, focusing on key factors that determine optimal locations for solar energy projects.

### Suitability analysis for photovoltaic installations in Mardan district

**Fig. 5:**
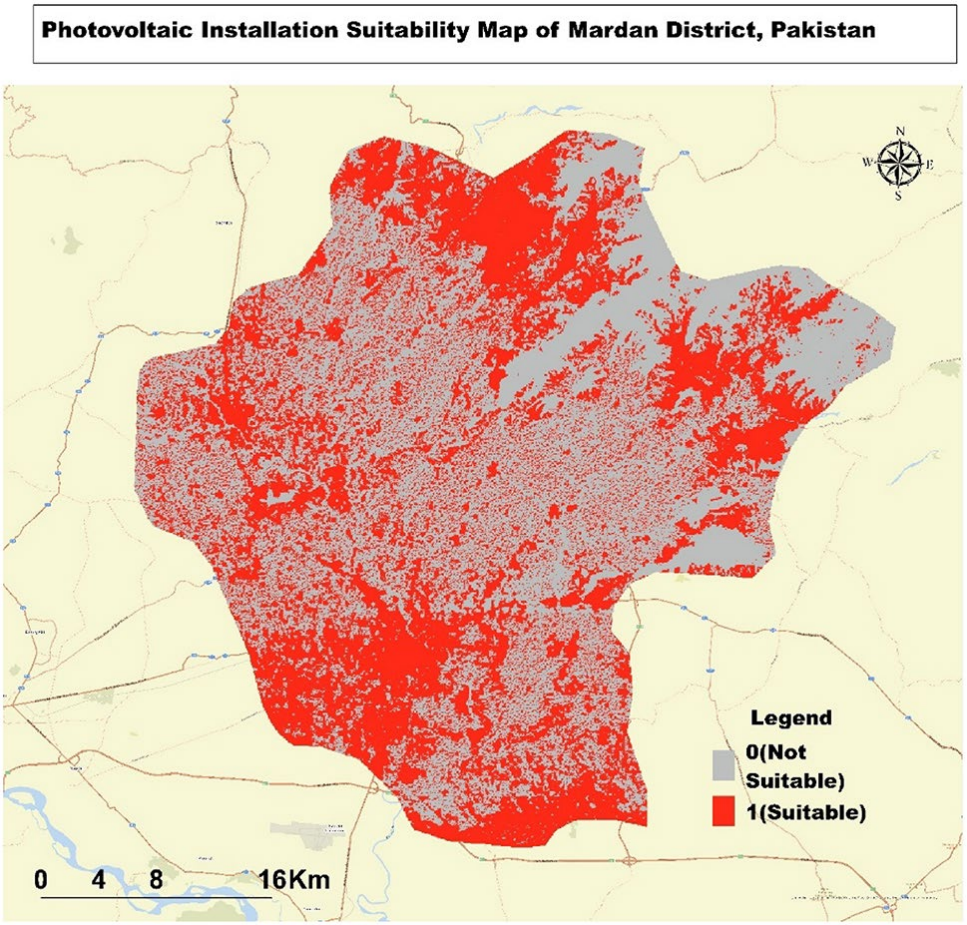
Suitability Map for Photovoltaic Installations in Mardan District: Highlighting Optimal and Less Suitable Areas.

The Map (Fig. 5 ) illustrates the suitability analysis for photovoltaic (PV) installation within the Mardan district of Pakistan. The central and western parts of Mardan emerge as the most suitable locations for solar panel placement, predominantly highlighted in red. These regions likely feature flatter terrain with ample sun exposure, making them ideal for PV installations. In contrast, the eastern and northeastern regions, where the presence of gray areas increases, indicate lower suitability. This corresponds to more mountainous terrain, where factors such as shading from hills, steeper slopes, or challenging access may hinder the feasibility of PV installations.

The city of Mardan and its immediate surroundings are also highlighted in red, underscoring the potential for both rooftop and ground-mounted solar installations in these populated areas. Additionally, major roads, such as the Mardan-Swabi Road and Katlang Road, are predominantly red, suggesting that the proximity to these routes could facilitate easier transportation and installation of PV equipment.

The areas around river valleys are similarly marked in red, suggesting that these lowland regions are suitable for PV installations. However, the northwestern border, particularly along the Malakand Road, shows a mix of red and gray, reflecting varying degrees of suitability as the terrain transitions. Flatter, more accessible areas closer to infrastructure appear to be favored for PV installation.

### Suitability analysis for photovoltaic installations in Nowshera district

**Fig. 6:**
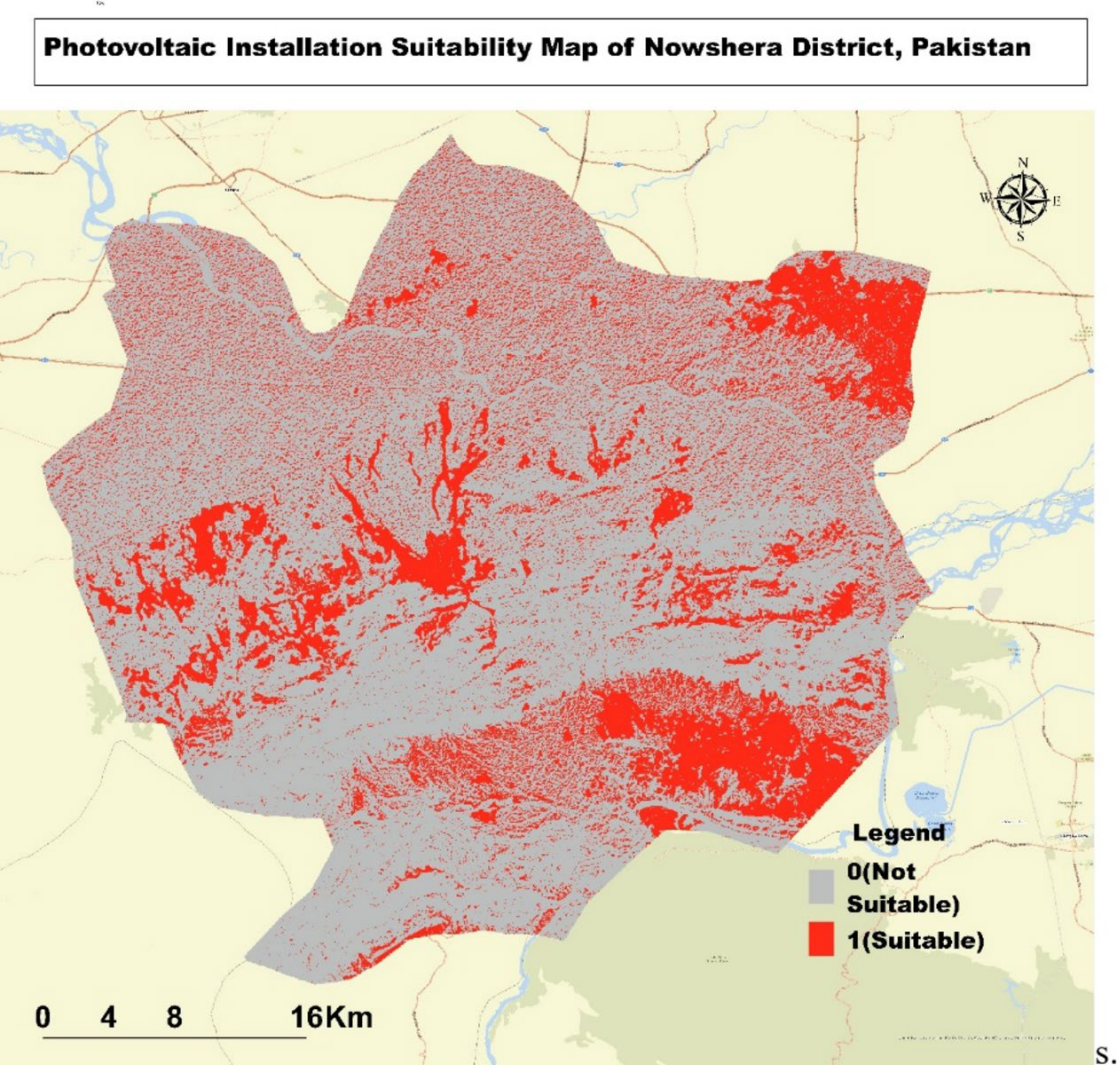
Suitability Map for Photovoltaic Installations in Nowshera District: Highlighting Optimal and Less Suitable Areas.

The Map (Fig. 6) illustrates the suitability analysis for photovoltaic (PV) installation in the Nowshera District of Pakistan. The areas highlighted in red indicate suitable locations for PV installation, while the gray areas are deemed unsuitable. Larger clusters of suitable areas are found predominantly in the eastern and southern regions, while the southwestern part, with its more rugged terrain, shows fewer viable locations. The eastern region, in particular, stands out with a notable concentration of suitable areas, suggesting favorable conditions for solar energy development. In contrast, the central and northern regions display a mix of suitable and unsuitable sites, likely influenced by local environmental factors. Interestingly, the proximity to rivers does not significantly impact the suitability, as optimal areas are scattered both near and distant from these water bodies. The Map, covering approximately 40–50 km across the district, underscores the importance of considering localized conditions in identifying the best sites for PV installations.

### Suitability analysis for photovoltaic installations in Peshawar district

**Fig. 7:**
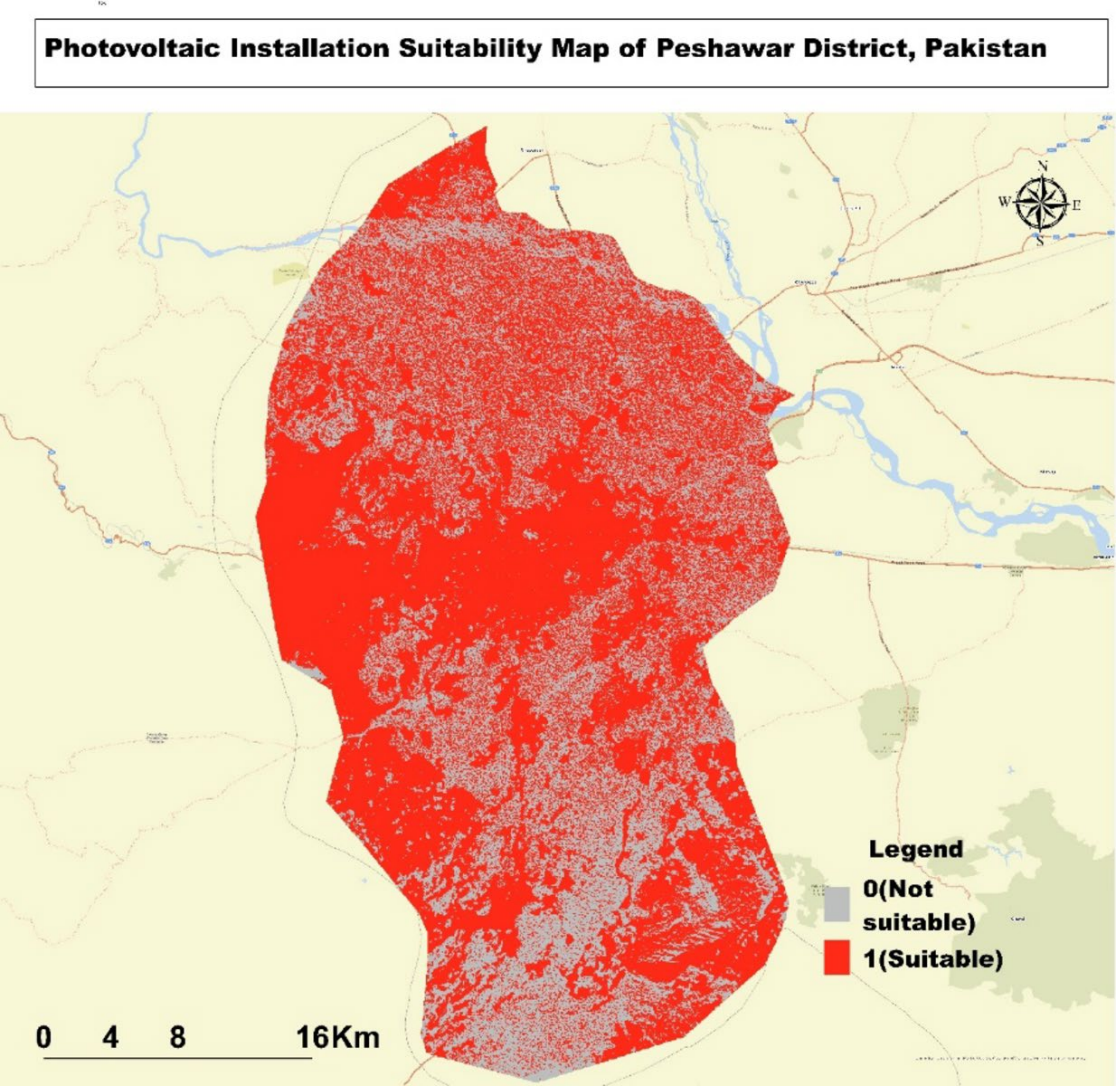
Suitability Map for Photovoltaic Installations in Peshawar District: Highlighting Optimal and Less Suitable Areas.

The Map **(**Fig. 7**)** showcases the suitability analysis for photovoltaic (PV) installations in Peshawar District, Pakistan. Red areas on the Map denote locations deemed suitable for PV installation, while gray regions within the red zones are identified as less favourable. A substantial portion of the district is highlighted in red, indicating that most areas are suitable for solar energy projects. However, the western edge of the district, characterized by rugged terrain, is excluded from the suitable areas, likely due to challenging slopes or shading effects. In contrast, the central and eastern regions exhibit a high concentration of suitable locations, corresponding to flatter terrain conducive to PV installations. Although major rivers and water bodies are visible on the Map, they do not significantly affect the suitability assessment, as suitable areas extend up to their banks. The central region, which likely includes Peshawar city, suggests strong potential for both urban and peri-urban solar installations. The Map’s scale, covering approximately 40–50 km at its widest point, emphasizes the extensive area available for solar development.

## Area distribution for PV suitability

In this section, the area distributions for photovoltaic (PV) suitability are provided for each region. The data details the total area covered by suitable and not suitable categories for each region Table 11 (a, b, c).

**Table 11:** Area distribution for PV suitability in (a- Mardan, b-Nowshera, c-Peshawar).

Object	Area(km²)	Count
(a)		
Not-Suitable	1,79,673	724.85,
Suitable	2,05,029	828.41
(b)		
Not Suitable	14,91,695	1,341.80
Suitable	5,60,429	503
(c)		
Not Suitable (0)	399.5	4,43,940
Suitable (1)	859.8	9,55,580

By examining the total areas classified as suitable for PV installations in each region, we can better assess their solar energy potential and identify regions with favorable conditions for future solar projects. It provide a detailed breakdown of the suitable areas within each region, offering insights into their respective capacities for supporting solar energy infrastructure.

### Energy output estimates

In the suitable areas of study areas, the energy output from photovoltaic (PV) installations was estimated using a fixed efficiency and average solar irradiance. The calculations were based on typical panel efficiency (18%) and the respective areas of the regions Table 12.

**Table 12:** Summarizes the energy output metrics for the three regions:.

Region	Metric	Value
Mardan	Average Solar Irradiance	1001.53 kWh/m²/day
	Daily Energy Output (avg)	108,835,650,155 kWh/day
	Annual Energy Output (avg)	39,736,040,708,575 kWh/year
Peshawar	Average Solar Irradiance	1195.71 kWh/m²/day
	Daily Energy Output (avg)	184,679,251,000 kWh/day
	Annual Energy Output (avg)	67,769,640,715,000 kWh/year
Nowshera	Average Solar Irradiance	1218.87 W/m²
	Daily Energy Output (avg)	1,835,809,395 kWh/day
	Annual Energy Output (avg)	670,057,137,675 kWh/year

For Mardan, the average solar irradiance was 1001.53 kWh/m²/day, with an estimated daily energy output of 108,835,650,155 kWh and an annual energy output of 39,736,040,708,575 kWh. Peshawar showed higher energy output, with an average daily output of 184,679,251,000 kWh and an annual output of 67,769,640,715,000 kWh. Nowshera exhibited the highest daily energy output of 1,835,809,395 kWh and an annual energy output of 670,057,137,675 kWh. These estimates assume fixed panel efficiency and optimal operating conditions.

### CO₂ Emissions from photovoltaic solar panels (Mardan, Peshawar, Nowshera)

Table 13 consolidates the data for energy output and CO₂ emissions across the three regions, providing a clear comparison. Mardan, with an average solar irradiance of 1001.53 W/m², generates a daily energy output of 108,835,650,155 kWh and an annual energy output of 39,736,040,708,575 kWh. Peshawar, benefiting from a higher average solar irradiance of 1195.71 W/m², achieves a larger daily energy output of 184,679,251,000 kWh and an annual output of 67,769,640,715,000 kWh. Nowshera, with the highest average solar irradiance at 1218.87 W/m², produces a lower daily energy output of 1,835,809,395 kWh and an annual output of 670,057,137,675 kWh.

**Table 13:** The annual energy output, emission factor, and total CO₂ emissions for Mardan, Peshawar, and Nowshera regions:.

Region	Annual Energy Output (kWh/year)	Emission Factor (g CO₂/kWh)	Total CO₂ Emissions (metric tons/year)
Mardan	39,736,040,708,575	41	1,628,177.67
Peshawar	67,769,640,715,000	41	2,779,626,309
Nowshera	670,057,137,675	41	27,463,344.975

The CO₂ emissions generated by photovoltaic (PV) solar panels can be calculated using the following formula: Eq. 12.

12 $$\:\begin{array}{c}\:{CO}_{2}\:\left(metric\:tones\right)=\frac{{E}_{output}{\left(\frac{kWh}{year}\right)\times\:E}_{emission}\left(\frac{\text{g}\text{C}\text{O}_{2}}{kWh}\right)\:}{100000}\end{array}$$

The Energy Output (kWh/year) represents the total annual energy generated by the photovoltaic (PV) panels. The Emission Factor refers to the amount of CO₂ emitted per kilowatt-hour (kWh) of electricity generated by the PV panels, with a value of 41 g of CO₂ per kWh. This factor is used to quantify the environmental impact of the PV system in terms of CO₂ emissions.

#### Comparative analysis of daily energy output and CO2 emissions in study area

**Fig. 8:**
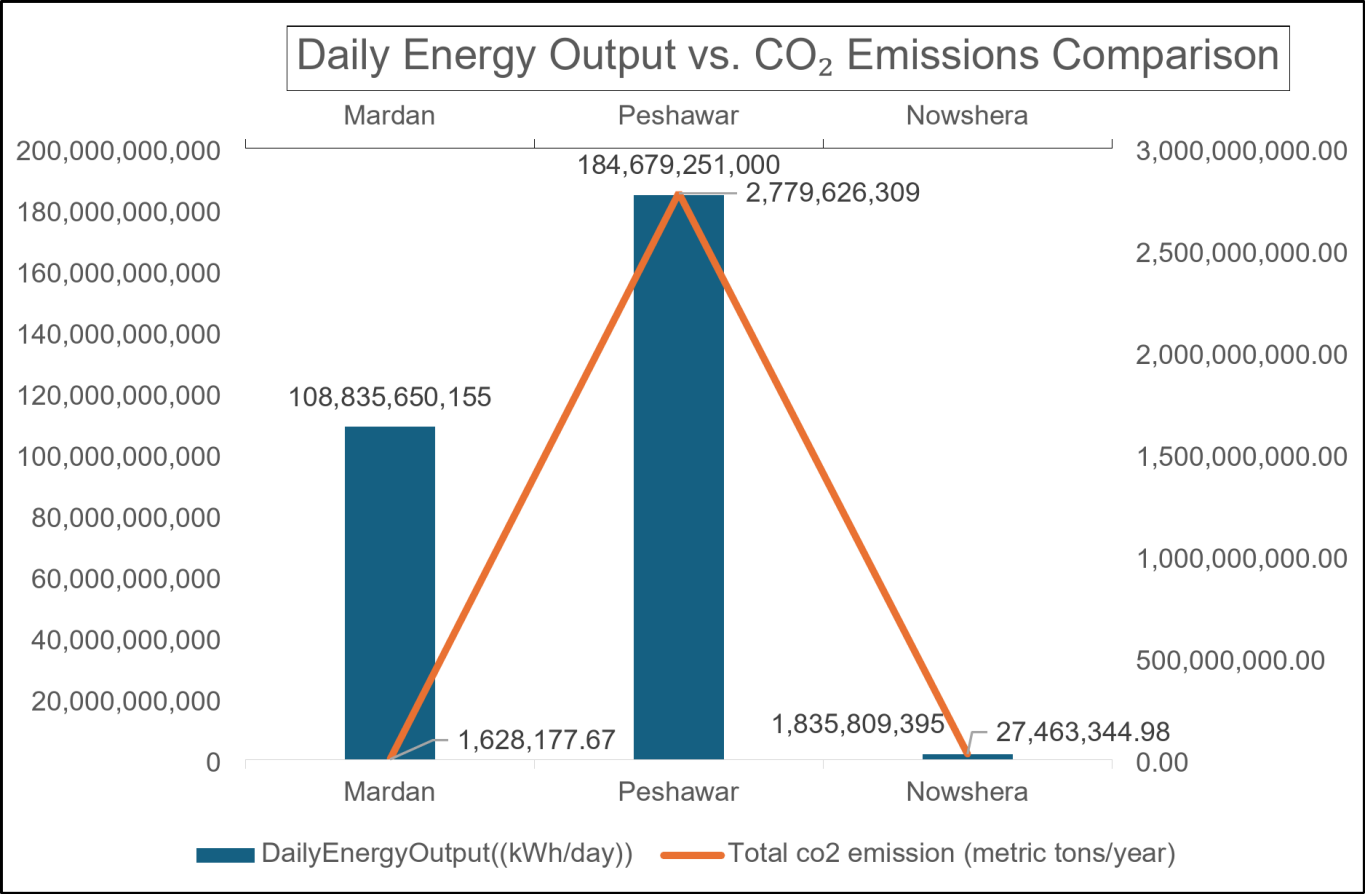
Comparative Analysis of Daily Energy Output and CO2 Emissions in Mardan, Peshawar, Nowshera.

Figure 8 illustrates the comparative analysis of daily energy output and associated CO_2_ emissions for the districts of Mardan, Peshawar, and Nowshera, highlighting significant regional variations in energy production and environmental impact.

The data reveals striking disparities in energy production and associated CO_2_ emissions across the three districts. Peshawar emerges as the leading energy producer, generating 184,679,251,000 kWh/day, followed by Mardan at 108,835,650,155 kWh/day, while Nowshera’s output is significantly lower at 1,835,809,395 kWh/day. Interestingly, the CO_2_ emissions do not strictly correlate with energy output. Peshawar, the highest energy producer, also emits the most CO_2_ at 2,779,626,309 metric tons/year. However, Mardan’s emissions are disproportionately low at 628,177.67 metric tons/year given its high energy output, suggesting potentially cleaner energy sources. Nowshera’s low energy production is matched by low emissions of 27,463,344.98 metric tons/year. These variations warrant further investigation into the energy sources, industrial activities, and potential efficiency measures in each district, as they have significant environmental and economic implications.

## Discussion

This study takes a unique approach to exploring the optimization of photovoltaic (PV) sites in the Mardan, Peshawar, and Nowshera districts. It utilizes advanced geospatial techniques and remote sensing data, making a significant contribution to the understanding of solar energy potential in these regions. This research has substantial implications for future energy planning and environmental management practices.

For the current analysis, significant topographical factors such as slope, aspect, viewshed, hillshade, geographic landforms were extracted using USGS DEM. In both scientific and empirical literature, these factors have been shown to be important for solar potential^28,58,59^. To classify land use and land cover (LULC), Sentinel-2 imagery was processed using the Random Forest Algorithm (RFA), which has proven effective in identifying areas suitable for energy production^60,61^.

Additionally, Land Surface Temperature (LST) was obtained from Landsat 9 data using Top of Atmosphere reflectance and NDVI to assess thermal conditions that can impact photovoltaic (PV) efficiency^62^. Prior studies have demonstrated the importance of accounting for temperature variations to evaluate PV performance^63,64^.

Incorporating Land Surface Temperature (LST) data into this study provides a unique and valuable approach for improving the accuracy of solar energy performance estimates. LST is an important factor that affects the performance of photovoltaic (PV) systems because excessive temperature decreases the energy produced by solar modules. This study incorporates local temperature differing effects, important for local performance of solar panels, by embedding LST data. This approach enables more accurate forecasting of solar energy production, as it incorporates not only general solar irradiance but also the specific effect of temperature on the efficiency of the PV [photo-voltaic] system.

In this study, the use of LST data is particularly novel, as it helps us better understand the recent environmental context of solar radiation − that is, how land surface temperature can result in more localized and dynamic understanding of the environmental variables that affect solar power plants. Although most previous studies use only solar radiation data, LST can specifically represent the real-time thermal status of a solar panel, which results in more accurate and reliable prediction of energy generation. Including land surface temperature (LST) improves solar energy estimates and enables improved solar siting criteria, ensuring that sites are not just sunny, but thermally suitable for energy production.

Parameters were reclassified into binary categories (suitable or unsuitable) based on empirical thresholds and expert input, particularly considering solar radiation and slope. This method, using the Weight Sum tool in ArcGIS Pro, created a composite map showing solar suitability^35,65^.The use of Multi-Criteria Decision Analysis (MCDA) in GIS for renewable energy site selection is well established^28,66^.

Suitability maps for PV installations in Mardan show that central and western regions with flatter terrain and optimal sunlight are more suitable than the eastern areas with steep slopes and shading, emphasizing the importance of local topography in site selection. This aligns with findings from various studies that emphasize the critical role of terrain and shading in determining PV site suitability. For instance, research in Jamaica highlighted the importance of geographic and environmental factors in site selection for solar farms, identifying suitable areas based on global horizontal irradiance and topography [30]. Similarly, studies in Inner Mongolia and the Palestinian territories utilized the Analytic Hierarchy Process (AHP) to assess land suitability, confirming that topographic features significantly influence solar energy potential^67,68^. These findings collectively underscore the necessity of considering local geographic conditions when evaluating PV installation sites to optimize energy generation.

GIS-based analyses have also been shown to be important in the PV site selection process in prior studies. For instance^52^emphasized land use and slope topographical and technical criteria in Algeria Building on this^35^, using the Multi-Influencing Factor (MIF) technique, conducted a GIS-based analysis in the Nashik region of India, improving the accuracy of identifying optimal sites for solar PV farms by considering factors such as solar radiation, wind speed, Land Surface Temperature (LST), relative humidity, and more, achieving an accuracy of 81.80% in predicting suitable sites. Similarly^55^, employed GIS and Multi-Criteria Decision Making (MCDM) method was used to analyze the potential sites for solar and wind farm all over India. Studies of^69^showed the importance of cloud cover for irradiance, which is in accordance with our Mardan case, whilst microclimatic effect on energy output was also validated. Findings from Jamaica^39,70^ has also highlighted the role topography and geographic variables; our results show that less terrain undulation and less shading are important for PV deployment.

Unlike^58,71^, that focused only on solar radiation and land use, our incorporation of LST provides the precision of suitability maps. These comparisons emphasize the benefit of using various inputs datasets and local specific parameters in order to adjust for optimal PV site selection on a regional scale.

The suitability map of Nowshera shows that the region in the eastern and southern parts is ideal for the installation of photovoltaic systems (PV) because these regions have flatter topography with better sunshine due to east-west orientation conforms to every other, revealing significant importance of terrain conditions solar energy planning^28^. Similarly, the minimal shading and favorable topography of Central and East Peshawar indicate that site selection would be key to achieving the highest solar energy potential^24^. In contrast, the rugged western regions are less suitable, a limitation echoed in assessments of solar energy in mountainous areas, which often face similar topographical challenges^72^. Such integration of geographic information systems and multi-criteria decision-making frameworks in these studies demonstrate the importance of incorporating both environmental as well as economic aspects into solar energy site-suitability analysis, which will help to develop more actionable intelligence for different agents, including governments, investment bodies, leaders (of either private or public sectors) involved in planning such projects over a variety level plane^24^^28,72^.

Significant regional differences in comparing photovoltaic (PV) energy outputs and CO₂ emissions are observed by provinces, i.e., Peshawar, Nowshera, and Mardan. Peshawar demonstrates an annual potential of about 67.77 trillion kWh/year, indicating its substantial potential for solar energy production, while Nowshera and Mardan follow with lower outputs and emissions^73^. This variation is strongly related to solar incidents and PV’s surface need, the main drivers determining electricity generation^6,24^. Importantly, higher energy released by Peshawar has more CO₂ emissions than lower but opposite features acting in Nowshehra and Mardan. Their respective have the lowest CO₂ emission results. This study also ties in with larger investigations that assert the significance of local impacts on energy production and environmental havoc^74,75^. The use of renewable energy sources in these districts serves as an example for reducing carbon footprints and yet feeding the increasing demands.

Comparison of daily energy output and CO₂ emissions across Mardan, Peshawar, and Nowshera. Its considerably larger energy output compared to Peshawar (184,679,251,000 kWh/day) results in the greatest CO₂ emissions. Due to Mardan emitting considerably less than similarly powerful energy sources, it points towards this possibly cleaner fuel source that deserves more research. These results reflect the paradox of various aspects of energy production versus impact within this region.

A mathematical model was also used to correct the energy output estimates accordingly by accounting for how temperature affects the seasonal production of a standard PV panel. This model calibrates the real power output (Ppv) to local meteorological conditions, particularly surface temperature. Additionally, the model proposed by Ilboudo et al. emphasizes the correlation between sunlight and ambient temperature, leading to a formula that calculates “temperature-induced efficiency” for PV modules, which varies by location (e.g., efficiencies of 0.9 to 0.98 in different cities^76^. This understanding is further enhanced by the additional study in^77^that provides models to predict module temperatures very accurately under various climatic conditions, with high accuracy (R² > 0.91). The study^78^confirms a thermal model that estimates cell temperatures and power outputs in excellent agreement with experiential data. The research in^79^presents an innovative method for power prediction based on 7 parameters by incorporating temperature and irradiance fluctuations, which provides the highest accuracy. Collectively, these conclusions illustrate the utility of using site-specific meteorological information in PV performance models to improve the quantification of energy yield. The formula (Eq. 10, Eq. 11)^23,80,81^, calculates the actual power of a photovoltaic module under real-life operating conditions.

## Calculate real power output

Using the formula: Eq. 13

13 $$\:\begin{array}{c}{P}_{pv}\:={P}_{STC\:}[\:1+{\beta\:}_{\rho\:}({T}_{cell-\:}{T}_{STC\:}\left)\right]\frac{{H}_{t}}{{H}_{STC}}\end{array}$$

## Calculate cell surface temperature [T_cell_]:

Using the empirical formula: Eq. 14

14 $$\:\begin{array}{c}{T}_{cell\:\:}={\:T}_{\infty\:}+7.8\:\times\:\:{10}^{-2}{H}_{t}\end{array}$$

Where [$\:{P}_{pv}$] is the real power of the PV module based on actual operating conditions. $\:{P}_{STC\:}$ is the power at Standard Test Conditions. ​ $\:{\beta\:}_{\rho\:}$​ is the power temperature coefficient [in W/°C], indicating the rate at which power output decreases with increasing temperature. ​$\:{T}_{STC\:}$​ is the cell’s surface temperature at Standard Test Conditions [in °C]. ​$\:{H}_{STC}$ is the standard solar radiation [in W/m²]. $\:{H}_{t}$ is the real solar radiation incident on the module [in W/m²]. $\:{T}_{cell\:\:}$ ​ is the surface cell temperature (in °C).

The formula adjusts power output for real conditions, revealing that higher temperatures in Mardan, Peshawar, and Nowshera may reduce PV efficiency, offering a more realistic estimate of energy production. These adjustments highlight the importance of considering local environmental factors, beyond the average solar irradiance, when estimating the energy production potential of PV systems.

### Implications and recommendations

The research highlights the need to plan solar energy projects specific to the region. Peshawar is presented as the city with the highest potential for photovoltaic (PV) and especially, for rooftops solar with its significantly larger developments land areas. Mardan and Nowshera are also areas full of potential but with the differing challenges and opportunities they present for solar deployment. The analysis shows that solar PV technology will be key in future energy systems and highlights the importance of finding the right location to deploy it.There are a lot of advantages to harnessing solar energy, such as to lessen the reliance on non-renewable energy and the harm that the use of fossil fuels do to the environment. But these advantages can only be maximized via deliberate, customized site selection.

It advocates the use of similar selection criteria in future solar facilities to balance deployment speed with sustainability over time. We need to start with fixing the weaknesses that have caught on in Nowshera, while it may not be all fit for solar energy generation. It is necessary to have adaptive technologies and better planning practices to make them more appropriate. Both least and most favourable areas are within the city boundaries of Mardan, so our task here is to strike a balance between optimally using the least desirable areas and maximally useable land within the most favourable regions where it is available. Peshawar, with its abundance of land with enables scaling, spread across the territory is an enormously colossal opportunity for large scale solar power projects, driving and changing over intotherenewable energy.

This study can serve as a proxy for local policymakers to understand the effect of different geographic and climatic factors in their respective regions on solar energy potential. Policymakers could apply this data to create better energy policies that would prioritize the development of solar power systems in locations where they will be most productive. Identifying the lead regions revealed by this analysis facilitates better site-selection decisions to stimulate a solar energy transition while maximizing the co-benefits of renewable adaptation to land. Not only can this type of localized planning help to lead future policy and financial interests to help facilitate solar energy growth, but also can be implemented in a way making the increased solar energy growth feasible in the long-term.

The inclusion of local-scale data including Land Surface Temperature (LST) and the explicit suitability analysis would further facilitate the energy planners for designing and developing effective PV systems based on the performance metrics. This information can help planners generate improved energy production forecasts and more accurately direct investments towards solar infrastructure. Energy planners will ensure that solar projects that impact both local temperature and the land can be tailored for cost-effectiveness and resiliency. This study may serve as method for future solar energy planning to guarantee that the performances and costs of solar energy projects are well adopted.

The study provides information to the investors regarding energy generation potential using solar projects in Peshawar, Mardan and Nowshera city. With LST data and other local variables, one can better visualize the energy production of solar panels in these areas, hence, investors can estimate solar energy output more accurately. By providing a more accurate outlook of where energy can be produced, this improved forecast mitigates the risks of investing in solar projects, directing resources to places that can yield the best-performing solar power sites. In addition, the insights can help investors discover growth potential in the expanding renewable energy market and accelerate the shift toward sustainable energy while ensuring that investors capture the long-term value of their investments.

Overall, this study highlights the importance of regional planning when deploying solar PV systems. These findings should be taken into account in future solar implementations as they can help to better meet local needs and can also identify potential opportunities that can be leveraged in each region. Such methods would make sure that solar power projects have been efficient & efficient, but economically viable & aligned with wider environmental objectives.

#### Limitations and assumptions of the study

While the study provides valuable insights into photovoltaic (PV) site selection in Mardan, Peshawar, and Nowshera, several limitations must be considered in interpreting the findings.

One limitation is the resolution and accuracy of the remote sensing data used in the analysis. The Sentinel-2 and Landsat imagery, while effective, might not fully capture fine-scale variations in topography or land use. Higher resolution data could provide more precise results, but such data was not available or feasible within the scope of this study. Additionally, the accuracy of datasets such as digital elevation models (DEMs) and land use/land cover (LULC) classifications may be affected by temporal or spatial variations in data acquisition, potentially introducing some errors in the analysis.

Another limitation is the temporal aspect of the data. The study relies on data from a specific time frame, which might not fully account for seasonal or long-term variations in environmental conditions. For instance, solar radiation and temperature fluctuate throughout the year, and such variations could affect PV efficiency, but were not captured in the static dataset used.

The assumptions made in the study regarding key parameters could also introduce uncertainty. For example, the temperature coefficients for PV panels and the reclassification thresholds used in the suitability analysis were derived from literature and expert input. These values may not be universally applicable to all locations or climate conditions, and variations in these assumptions could influence the results.

Moreover, the study simplifies the classification of land suitability into binary categories—suitable or unsuitable—which may overlook the complexity of real-world site selection. A more nuanced approach could consider factors such as socio-economic conditions, grid connectivity, and broader environmental concerns like biodiversity, which were beyond the scope of this research but are crucial in practical PV site selection.

Additionally, while expert input was used to define reclassification thresholds, this could introduce bias. A wider range of stakeholder inputs, including those from local communities and policymakers, could enhance the robustness of the results and ensure more balanced decision-making.

The geographic scope of the study is also a limitation. While the methodology is applicable to other regions, the specific findings are tied to the environmental and climatic conditions of Mardan, Peshawar, and Nowshera. For application to other areas, adjustments to the methodology would be necessary to account for differences in local conditions.

Lastly, the study primarily focuses on physical and environmental factors in PV site selection, but it does not consider broader environmental or social impacts, such as effects on ecosystems, land rights, or community acceptance. These factors play an important role in the feasibility and sustainability of PV projects and should be taken into account in future research.

In conclusion, while this study makes a significant contribution to PV site selection in the region, it is essential to acknowledge these limitations to better understand the scope of the results and to guide future studies in overcoming these challenges.

## Conclusion

This study provides a comprehensive framework for optimizing photovoltaic (PV) energy installations in Mardan, Peshawar, and Nowshera through the integration of advanced geospatial and multi-criteria decision analysis techniques. The results emphasize the significant potential for solar energy deployment in these regions, with key factors such as solar radiation, terrain, and land use being pivotal in identifying the most suitable locations for PV installations.

The findings reveal that the central and southern parts of Mardan, eastern and southern areas of Nowshera, and extensive regions in Peshawar have the highest potential for solar energy generation, with Peshawar standing out due to its superior solar irradiance, leading to the highest annual energy output and CO₂ reductions. These regional differences underscore the importance of targeting areas with the greatest solar potential for large-scale PV deployment.

The study also offers actionable recommendations for policymakers, including prioritizing investments in high-potential areas, enhancing regional policies to encourage private sector involvement, promoting the adoption of photovoltaic technology, and integrating solar solutions into broader energy plans. By leveraging these insights, stakeholders can accelerate the transition to clean, renewable energy, improving energy security, reducing CO₂ emissions, and fostering long-term sustainability. This research not only contributes to the body of knowledge on renewable energy but also provides a replicable approach for optimizing solar energy deployment across diverse regions, benefiting both the environment and the local economy.

## Data Availability

The authors confirm that the datasets used and that support the findings of this study, are publicly available, derived from the following resources available in public domain (https://earthexplorer.usgs.gov/; https://scihub.copernicus.eu/). All sources are cited in the text and generated data, which is further analyzed is available within the article.

## References

[1] Naz, J., Ahmed, M. F. & Khan, R. A. Pakistan Energy Outlook for Next 25 years. *Bull. Bus. Econ. (BBE)*. **13** (2), 563–572 (2024).

[2] Chughtai, A., Uqaili, M. A., Hussain Mirjat, N., Shaikh, F. & Khatri, S. A. Demand-side management scenario analysis for the energy-efficient future of Pakistan: bridging the gap between market interests and national priorities. *Front. Energy Res.* **12**, 1391973 (2024).

[3] Khurshid, J., Khurshid, N. & Shaheen, S. *An Exploration of the Roots of a Chronic Energy Crisis in Pakistan. Energy Crisis and Its Impact on Global Business*p. 1–18 (IGI Global, 2024).

[4] Aized, T., Rehman, S. M. S. & Sumair, M. *Pakistan Energy Situation, Policy, and Issues. Recent Advances in Renewable Energy Technologies*p. 387–428 (Elsevier, 2021).

[5] Statista, R. D. Power production breakdown in Pakistan 2023, by source. Statista2024.

[6] Umer, M., Abas, N., Rauf, S., Saleem, M. S. & Dilshad, S. GHG Emissions Estimation and Assessment of Pakistan’s Power Sector: A Roadmap Towards Low Carbon Future. *Results Eng.* :102354. (2024).

[7] Iqbal, M., Ali, M., MATHEMATICAL APPROACH TO FORECAST & THE ELECTRICITY CRISES IN PAKISTAN AND ITS MITIGATION. *J. Mountain Area Res.* ;**9**:79–91. (2024).

[8] Filonchyk, M., Peterson, M. P., Zhang, L., Hurynovich, V. & He, Y. Greenhouse gases emissions and global climate change: examining the influence of CO2, CH4, and N2O. *Sci. Total Environ.* :173359. (2024). 10.1016/j.scitotenv.2024.173359 38768722

[9] Qudrat-Ullah, H. A review and analysis of renewable energy policies and CO2 emissions of Pakistan. *Energy* **238**, 121849 (2022).

[10] Mirzapour, O. & Arpanahi, S. K. (eds) Photovoltaic parameter estimation using heuristic optimization. IEEE 4th international conference on knowledge-based engineering and innovation (KBEI); 2017: IEEE. (2017).

[11] Energy USDo. Solar Futures Study. US Department of Energy [Internet]. (2023). Available from: https://www.energy.gov/eere/solar/solar-futures-study

[12] Thirunavukkarasu, M. & Sawle, Y. A comparative study of the optimal sizing and management of off-grid solar/wind/diesel and battery energy systems for remote areas. *Front. Energy Res.* **9**, 752043 (2021).

[13] Verma, D., Midtgård, O-M. & Sætre, T. O. (eds) Review of photovoltaic status in a European (EU) perspective. 2011 37th IEEE Photovoltaic Specialists Conference; : IEEE. (2011).

[14] Nassar, Y. et al. Assessing the viability of solar and wind energy technologies in semi-arid and arid regions: a case study of Libya’s climatic conditions. *Appl. Solar Energy*. **60** (1), 149–170 (2024).

[15] Alghanem, H. & Buckley, A. Global benchmarking and modelling of Installed Solar Photovoltaic Capacity by Country. *Energies* **17** (8), 1812 (2024).

[16] Solangi, Y. A. et al. An integrated Delphi-AHP and fuzzy TOPSIS approach toward ranking and selection of renewable energy resources in Pakistan. *Processes* **7** (2), 118 (2019).

[17] Abbas, Y. & Aslam, R. A. Potential of Untapped Renewable Energy Resources in Pakistan: Current Status and Future Prospects. Engineering Proceedings. ;56(1):108. (2023).

[18] RIAZ MN, PERDHANA MS. CHALLENGES IN IMPLEMENTING RENEWABLE ENERGY INITIATIVES IN PAKISTAN. UNDIP; Fakultas Ekonomika dan Bisnis; (2024).

[19] Muhammadi, A. et al. Solar Energy Potential in Pakistan: A Review. Proceedings of the Pakistan Academy of Sciences: B Life and Environmental Sciences. ;61(1):1–10. (2024).

[20] Xu, L., Wang, Y., Solangi, Y. A., Zameer, H. & Shah, S. A. A. Off-grid solar PV power generation system in Sindh, Pakistan: a techno-economic feasibility analysis. *Processes* **7** (5), 308 (2019).

[21] Solangi, Y. A., Shah, S. A. A., Zameer, H., Ikram, M. & Saracoglu, B. O. Assessing the solar PV power project site selection in Pakistan: based on AHP-fuzzy VIKOR approach. *Environ. Sci. Pollut. Res.* **26**, 30286–30302 (2019). 10.1007/s11356-019-06172-0 31432370

[22] Solangi, Y. A., Tan, Q., Mirjat, N. H. & Ali, S. Evaluating the strategies for sustainable energy planning in Pakistan: an integrated SWOT-AHP and Fuzzy-TOPSIS approach. *J. Clean. Prod.* **236**, 117655 (2019).

[23] Nassar, Y. F. & Alsadi, S. Y. Assessment of solar energy potential in Gaza Strip-Palestine. *Sustain. Energy Technol. Assess.* **31**, 318–328 (2019).

[24] Abid, M. A., Zhiyong, L., Shuai, M. & Rehman, M. S. U. (eds) MW Scale Grid-Connected Photovoltaic System Design Using GIS for Pakistan. IEEE 2nd International Conference on Power Science and Technology (ICPST); 2024: IEEE. (2024).

[25] Sheikh, N., Laverge, J. & Delghust, M. A Critical Analysis of Institutional and Regulatory Framework for Building Stock Energy Efficiency and Transition in Pakistan. *Environ. Sci. Sustainable Dev.* :32–41. (2024).

[26] Saleem, L., Ulfat, I. & Energy Society and Sociodemographic constraints Nexus: development and future prospects. *Sir Syed Univ. Res. J. Eng. Technol.* **14** (1), 07–11 (2024).

[27] Khan, N., Elhindi, K. M., Kassem, H. S., Kazim, R. & Zhang, S. Unveiling the nexus between solar energy adoption and crop farmer income: evidence from Pakistan. *Front. Sustainable Food Syst.* **8**, 1364040 (2024).

[28] Tryphena, B. & Vidhya, J. (eds) Enhancing Solar PV Deployment: Land Suitability Assessment and Site Selection using AHP. 2024 International Conference on Advances in Modern Age Technologies for Health and Engineering Science (AMATHE); : IEEE. (2024).

[29] Georgiou, A. & Skarlatos, D. Optimal site selection for sitting a solar park using multi-criteria decision analysis and geographical information systems. *Geoscientific Instrum. Methods Data Syst.* **5** (2), 321–332 (2016).

[30] Richards, D. et al. Sustainable solar energy deployment: a multicriteria decision-making approach for site suitability and greenhouse gas emission reduction. (2024). 10.1007/s11356-024-35669-6 39754625

[31] Islam, M. R., Aziz, M. T., Alauddin, M., Kader, Z. & Islam, M. R. Site suitability assessment for solar power plants in Bangladesh: a GIS-based analytical hierarchy process (AHP) and multi-criteria decision analysis (MCDA) approach. *Renew. Energy*. **220**, 119595 (2024).

[32] Shi, L. & Chew, M. Y. L. A review on sustainable design of renewable energy systems. *Renew. Sustain. Energy Rev.* **16** (1), 192–207 (2012).

[33] Ruiz, H., Sunarso, A., Ibrahim-Bathis, K., Murti, S. & Budiarto, I. GIS-AHP Multi Criteria Decision Analysis for the optimal location of solar energy plants at Indonesia. *Energy Rep.* **6**, 3249–3263 (2020).

[34] Suomalainen, K., Wang, V. & Sharp, B. Rooftop solar potential based on LiDAR data: bottom-up assessment at neighbourhood level. *Renew. Energy* ;**111**. (2017).

[35] Rane, N. L. et al. GIS-based multi-influencing factor (MIF) application for optimal site selection of solar photovoltaic power plant in Nashik, India. *Environ. Sci. Europe*. **36** (1), 5 (2024).

[36] Rafiq, L., Tajbar, S., Sahar, N. U. & Farid, F. Satellite data application for renewable energy: a comparative study in Khyber Pakhtunkhwa, Pakistan. *Arab. J. Geosci.* **14**, 1–8 (2021).

[37] Hadidi, A., Blal, M. & Saba, D. The study of the arid climate effect on the performance of photovoltaic system. *Energ. Syst.* :1–20. (2021).

[38] Avcı, C., Budak, M., Yağmur, N. & Balçık, F. Comparison between random forest and support vector machine algorithms for LULC classification. *Int. J. Eng. Geosci.* **8** (1), 1–10 (2023).

[39] Soni, P. K., Rajpal, N., Mehta, R. & Mishra, V. K. Urban land cover and land use classification using multispectral sentinal-2 imagery. *Multimedia Tools Appl.* **81** (26), 36853–36867 (2022).

[40] Amini, S., Saber, M., Rabiei-Dastjerdi, H. & Homayouni, S. Urban land use and land cover change analysis using random forest classification of landsat time series. *Remote Sens.* **14** (11), 2654 (2022).

[41] Junaid, M. et al. Mapping lulc dynamics and its potential implication on forest cover in malam jabba region with landsat time series imagery and random forest classification. *Sustainability* **15** (3), 1858 (2023).

[42] Zhang, Y., Ren, J., Pu, Y. & Wang, P. Solar energy potential assessment: a framework to integrate geographic, technological, and economic indices for a potential analysis. *Renew. Energy*. **149**, 577–586 (2020).

[43] Ku, J. & Park, H-D. Comparison of urban environment factors for Solar-Powered vehicles. *Int. Archives Photogrammetry Remote Sens. Spat. Inform. Sci.* **48**, 283–288 (2024).

[44] Gergelova, M. B. et al. Roof’s potential and suitability for PV systems based on LiDAR: a case study of Komárno. *Slovakia Sustain.* **12** (23), 10018 (2020).

[45] Merrouni, A. A., Mezrhab, A. & Mezrhab, A. PV sites suitability analysis in the eastern region of Morocco. *Sustain. Energy Technol. Assess.* **18**, 6–15 (2016).

[46] Günen, M. A. A comprehensive framework based on GIS-AHP for the installation of solar PV farms in Kahramanmaraş, Turkey. *Renew. Energy*. **178**, 212–225 (2021).

[47] Iqbal, M. *An Introduction to Solar Radiation* (Elsevier, 2012).

[48] Darabi, S., Monavari, S. M., Jozi, S. A., Rahimi, R. & Vafaeinejad, A. Visual impact assessment of renewable energy developments with the application of multi-criteria decision-making method. *Environ. Dev. Sustain.* **25** (5), 4437–4451 (2023).

[49] Merrouni, A. A., Elalaoui, F. E., Mezrhab, A., Mezrhab, A. & Ghennioui, A. Large scale PV sites selection by combining GIS and Analytical Hierarchy process. Case study: Eastern Morocco. *Renew. Energy*. **119**, 863–873 (2018).

[50] Jong, F. C. & Ahmed, M. M. Multi-criteria Decision-Making Solutions for Optimal Solar Energy Sites Identification: a systematic review and analysis. *IEEE Access.* (2024).

[51] Rinner, C. Spatial Dimensions of Multi-Criteria Analysis. (2024).

[52] Gairaa, K., Guermoui, M., Zaiani, M., Belaid, S. & Benkaciali, S. (eds) Optimal Land Suitability Based on GIS Tools for Solar PV Farms. 2023 8th International Conference on Smart and Sustainable Technologies (SpliTech); : IEEE. (2023).

[53] Al Garni, H. & Awasthi, A. Chapter 2-Solar PV power plants site selection: a review: (ed Imene, Y.) Advances in Renewable Energies and Power Technologies. Elsevier; (2018).

[54] Hashemizadeh, A., Ju, Y. & Dong, P. A combined geographical information system and best–worst method approach for site selection for photovoltaic power plant projects. *Int. J. Environ. Sci. Technol.* **17**, 2027–2042 (2020).

[55] Saraswat, S., Digalwar, A. K., Yadav, S. & Kumar, G. MCDM and GIS based modelling technique for assessment of solar and wind farm locations in India. *Renew. Energy*. **169**, 865–884 (2021).

[56] Kannan, D., Moazzeni, S., mostafayi Darmian, S. & Afrasiabi, A. A hybrid approach based on MCDM methods and Monte Carlo simulation for sustainable evaluation of potential solar sites in east of Iran. *J. Clean. Prod.* **279**, 122368 (2021).

[57] Saraji, M. K., Streimikiene, D. & Suresh, V. A novel two-stage multicriteria decision-making approach for selecting solar farm sites: a case study. *J. Clean. Prod.* **444**, 141198 (2024).

[58] Noorollahi, Y., Senani, A. G., Fadaei, A., Simaee, M. & Moltames, R. A framework for GIS-based site selection and technical potential evaluation of PV solar farm using fuzzy-boolean logic and AHP multi-criteria decision-making approach. *Renew. Energy*. **186**, 89–104 (2022).

[59] Brewer, J., Ames, D. P., Solan, D., Lee, R. & Carlisle, J. Using GIS analytics and social preference data to evaluate utility-scale solar power site suitability. *Renew. Energy*. **81**, 825–836 (2015).

[60] Zhou, X., Lu, P., Zheng, Z., Tolliver, D. & Keramati, A. Accident prediction accuracy assessment for highway-rail grade crossings using random forest algorithm compared with decision tree. *Reliab. Eng. Syst. Saf.* **200**, 106931 (2020).

[61] Badshah, M. T. et al. The role of random forest and markov chain models in understanding metropolitan urban growth trajectory. *Front. Forests Global Change*. **7**, 1345047 (2024).

[62] Meng, X., Cheng, J., Guo, H., Guo, Y. & Yao, B. Accuracy evaluation of the Landsat 9 land surface temperature product. *IEEE J. Sel. Top. Appl. Earth Observations Remote Sens.* **15**, 8694–8703 (2022).

[63] Karimi, A. & Ghajari, Y. E. Improving land surface temperature prediction using spatiotemporal factors through a genetic-based selection procedure (case study: Tehran, Iran). *Adv. Space Res.* **69** (9), 3258–3267 (2022).

[64] Mokhtari, M. H., Ahmadikhub, A. & Saeedi-Sourck, H. Substitution of satellite-based land surface temperature defective data using GSP method. *Adv. Space Res.* **67** (10), 3106–3124 (2021).

[65] Sun, Y., Zhu, D., Li, Y., Wang, R. & Ma, R. Spatial modelling the location choice of large-scale solar photovoltaic power plants: application of interpretable machine learning techniques and the national inventory. *Energy. Conv. Manag.* **289**, 117198 (2023).

[66] Sahin, G., Akkus, I., Koc, A. & van Sark, W. Multi-criteria solar power plant siting problem solution using a GIS-Taguchi loss function based interval type-2 fuzzy approach: the case of Kars Province/Turkey. *Heliyon* ;**10**(10). (2024). 10.1016/j.heliyon.2024.e30993 PMC11108993 38779030

[67] Liu, T. et al. Suitability analysis for implementing wind and solar farms based AHP method: Case study in Inner Mongolia, China. The International Archives of the Photogrammetry, Remote Sensing and Spatial Information Sciences. ;48:417 – 23. (2024).

[68] Qutaina, B., Shehada, A., Yasin, A. & Alsayed, M. Geographical information systems based site selection methodology for renewable energy systems in Palestinian territories. *Int. J. Electr. Comput. Eng.* (2088–8708). 2023;13(4).

[69] Wang, F. et al. A minutely solar irradiance forecasting method based on real-time sky image-irradiance mapping model. *Energy. Conv. Manag.* **220**, 113075 (2020).

[70] Yu, S., Han, R. & Zhang, J. Reassessment of the potential for centralized and distributed photovoltaic power generation in China: on a prefecture-level city scale. *Energy* **262**, 125436 (2023).

[71] Van de Ven, D-J. et al. The potential land requirements and related land use change emissions of solar energy. *Sci. Rep.* **11** (1), 1–12 (2021). 33536519 10.1038/s41598-021-82042-5 PMC7859221

[72] Hamad, J., Ahmad, M. & Zeeshan, M. Solar energy resource mapping, site suitability and techno-economic feasibility analysis for utility scale photovoltaic power plants in Afghanistan. *Energy. Conv. Manag.* **303**, 118188 (2024).

[73] Khattak, S., Yousif, M., Hassan, S. U., Hassan, M. & Alghamdi, T. A. Techno-economic and environmental analysis of renewable energy integration in irrigation systems: a comparative study of standalone and grid-connected PV/diesel generator systems in Khyber Pakhtunkhwa. *Heliyon* ;**10**(10). (2024). 10.1016/j.heliyon.2024.e31025 PMC11128857 38803921

[74] Hammas, S. & Khalili, A. Land Use and Energy Comparison of grid-connected Monocrystalline, and Heterojunction with intrinsic thin-layer Solar technologies using advanced PVsyst Software (A Case Study in Kabul Province, Afghanistan). *Ajrsp* **6** (63), 121–133 (2024).

[75] Khan, M. M., Ahmad, S., Tariq, M. U., Anjum, Z. H. & Shafi, M. A. Simulation design of 542kWp DC/480kWp AC Solar Photovoltaic System at Institute of Southern Punjab. *Sir Syed Univ. Res. J. Eng. Technol.* **14** (1), 102–107 (2024).

[76] Jacques Marie, I., Dominique, B. & Zacharie, K. A New Approach to sizing PV modules while Accounting the Effect of temperature. *Am. J. Energy Eng.* **11** (4), 127–133 (2023).

[77] Aissa, M., Aouchiche, I., Berkane, S. & Chekired, F. Estimation models of photovoltaic module operating temperature under various climatic conditions. *Indonesian J. Electr. Eng. Comput. Sci.* **32**, 13–20 (2023).

[78] Khyani, H. K., Vajpai, J., Karwa, R. & Bhadu, M. Thermal modeling of Photovoltaic Panel for Cell temperature and power output predictions under Outdoor climatic conditions of Jodhpur. *J. Electr. Comput. Eng.* **2023** (1), 5973076 (2023).

[79] Kumar, M., Malik, P., Chandel, R. & Chandel, S. S. Development of a novel solar PV module model for reliable power prediction under real outdoor conditions. *Renew. Energy*. **217**, 119224 (2023).

[80] Nassar, Y. F., Alsadi, S. Y., Miskeen, G. M., El-Khozondar, H. J. & Abuhamoud, N. M. (eds) Mapping of PV solar module technologies across Libyan territory. 2022 Iraqi International Conference on Communication and Information Technologies (IICCIT); : IEEE. (2022).

[81] Nassar, Y. F. et al. A new design for a built-in hybrid energy system, parabolic dish solar concentrator and bioenergy (PDSC/BG): a case study–Libya. *J. Clean. Prod.* **441**, 140944 (2024).

